# The Contribution of Neutral and Environmentally Dependent Processes in Driving Population and Lineage Divergence in Taiwania (*Taiwania cryptomerioides*)

**DOI:** 10.3389/fpls.2018.01148

**Published:** 2018-08-08

**Authors:** Yi-Shao Li, Chung-Te Chang, Chun-Neng Wang, Philip Thomas, Jeng-Der Chung, Shih-Ying Hwang

**Affiliations:** ^1^Department of Life Science, National Taiwan Normal University, Taipei, Taiwan; ^2^Department of Geography, National Taiwan University, Taipei, Taiwan; ^3^Institute of Ecology and Evolution, National Taiwan University, Taipei, Taiwan; ^4^International Conifer Conservation Programme of the Royal Botanic Garden, Edinburgh, United Kingdom; ^5^Division of Silviculture, Taiwan Forestry Research Institute, Taipei, Taiwan

**Keywords:** adaptive divergence, epigenetic variation, genetic variation, nonadaptive divergence, *Taiwania cryptomerioides*

## Abstract

The question of what determines divergence both between and within species has been the central topic in evolutionary biology. Neutral drift and environmentally dependent divergence are predicted to play roles in driving population and lineage divergence. However, neutral drift may preclude adaptation if the rate of gene flow between populations is high. Here, we sampled populations of three Taiwania (*Taiwania cryptomerioides*) lineages occurring in Taiwan, the mainland of China (Yunnan-Myanmar border), and northern Vietnam, and tested the relative strength of neutral drift and divergent selection in shaping divergence of those populations and lineages. We quantified genetic and epigenetic variation, respectively, using amplified fragment length polymorphism (AFLP) and methylation-sensitive amplification polymorphism (MSAP). Analysis of 1413 AFLP and 462 MSAP loci using frequency-based genome scan methods and generalized linear models (GLMs) found no potential selective outliers when only Taiwanese populations were examined, suggesting that neutral drift was the predominant evolutionary process driving differentiation between those populations. However, environmentally associated divergence was found when lineages were compared. Thirty-two potential selective outliers were identified based on genome scans and their associations with environmental variables were tested with GLMs, generalized linear mixed effect models (GLMMs), and model selection with a model averaging approach. Ten loci (six AFLP and four MSAP) were found to be strongly associated with environmental variables, particularly monthly temperature variation and normalized difference vegetation index (NDVI) using model selection and a model averaging approach. Because only a small portion of genetic and epigenetic loci were found to be potential selective outliers, neutral evolutionary process might also have played crucial roles in driving lineage divergence, particularly between geographically and genetically isolated island and mainland Asia lineages. Nevertheless, the vast amount of neutral drift causing genetic and epigenetic variations might have the potential for adaptation to future climate changes. These could be important for the survival of Taiwania in different geographic areas.

## Introduction

Both selective and neutral forces may be involved in population and lineage diversification. Elucidating their relative strength in driving biological variation is critical to understanding how these processes impact evolution at population level and early stages of speciation (Coyne and Orr, 2004; Rundell and Price, 2009; Raeymaekers et al., 2017). Adaptive lineages can evolve within the species distribution range when associated environmental gradients underlie local adaptation (Alberto et al., 2013; Savolainen et al., 2013). Environmentally associated genetic variation can be used as a stepping stone in identifying new ecotypes that can be useful in the future conservation of species. However, historical events and stochastic or neutral mechanisms can also play important roles in shaping the gene pool within the current distribution area (Lande, 1988; Wang et al., 2017).

Selection driven by ecological factors acting on genetic variation of DNA sequences is of major importance in evolutionary biology (Schluter, 2001, 2009). DNA sequence variation revealed by amplified fragment length polymorphism (AFLP) has been found to be closely associated with environmental conditions in shaping population adaptive divergence of many plant species (e.g., Fang et al., 2013; Huang et al., 2015a,b; Santiso et al., 2016; Yang et al., 2016; Chen et al., 2017). There is an increasing interest in investigating environmentally dependent epigenetic variation in natural populations, which is also important for understanding the potential for adaptation of populations and species that are enduring rapid global environmental changes (Bossdorf et al., 2008; Johnson and Tricker, 2010; Alonso et al., 2015; Huang et al., 2015b; Villota-Salazar et al., 2016). Such variation can be characterized by methylation-sensitive amplification polymorphism (MSAP) that reflects modification of cytosine methylation states (Bossdorf et al., 2008; Richards et al., 2017). The association between epigenetic variation and environments may be related to the lower levels of methylation status in different genes, resulting in the higher expression of fitness-related traits (Lira-Medeiros et al., 2010; Latzel et al., 2013; Whipple and Holeski, 2016; Richards et al., 2017). Three types of epigenetic variation (obligatory, facilitated, and pure epigenetic variation) have been characterized to explain the degree to which epigenetic and DNA sequence variation are related (Richards, 2006). In obligatory epigenetic variation, the epigenotype is entirely dependent on genotype. The relationship between epigenotype and genotype is either partially or completely independent in facilitated and pure epigenetic variation.

Taiwania (*Taiwania cryptomerioides* Hayata) is a coniferous species in the monotypic genus *Taiwania* of the cypress family Cupressaceae. While its fossil record indicates that it was formerly widespread across the northern Hemisphere (LePage, 2009), it currently has a disjunct distribution in Taiwan, northern Vietnam, and mainland China (Wang and Xie, 2004; Farjon and Thomas, 2007; Nguyen, 2007). Three main lineages have been identified in natural populations in Taiwan, along the Yunnan-Myanmar border, and in Vietnam (Chou et al., 2011). The Taiwanese and Yunnan-Myanmar lineages diverged between 3.23 and 3.41 million years ago (Mya), while Yunnan-Myanmar and Vietnamese lineages diverged between 1.0 and 1.39 Mya (Chou et al., 2011). The population located along the Yunnan-Myanmar border included four chloroplast DNA (cpDNA) haplotypes. The most common of these was also the only haplotype found in other doubtfully naturally occurring Chinese populations. The Vietnamese lineage contained five cpDNA haplotypes including the high frequency haplotype found in all mainland China populations and also shared one low frequency haplotype with Yunnan-Myanmar lineage. Only two cpDNA haplotypes, both different from those found in mainland China and Vietnam, were found in Taiwan. Overall, a low level of cpDNA variation was observed in Taiwania (Chou et al., 2011) in contrast to other related widespread cypress species (*Cunninghamia konishii* and *Cu. lanceolata*, Hwang et al., 2003).

**Figure 1:**
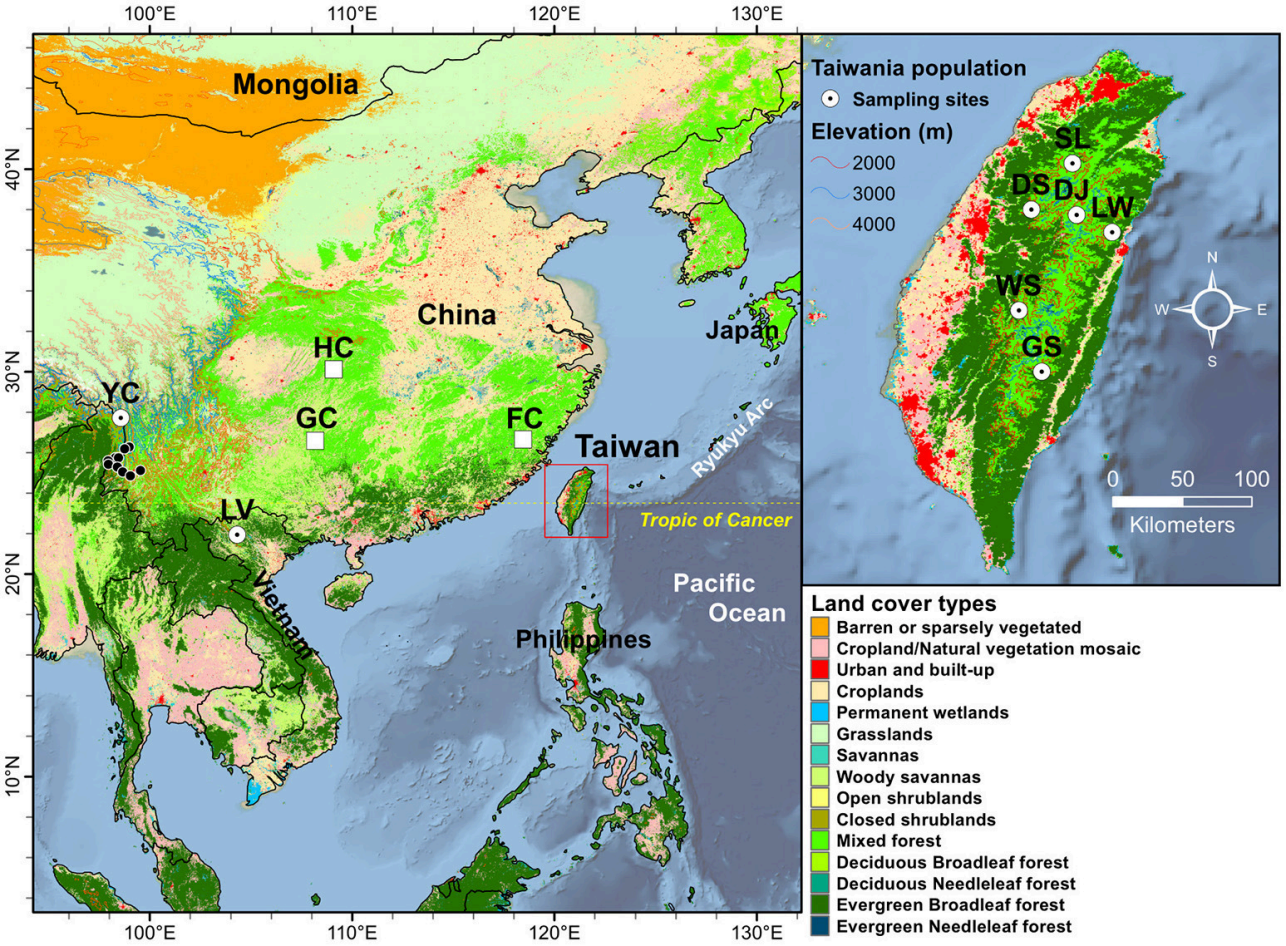
Geographic distribution of the eight populations of Taiwania in the Taiwanese, Yunnan-Myanmar, and Vietnamese lineages. Land cover types were extracted using MODIS product MCD12Q1 of 2013 at 500 m resolution. See Table 1 for abbreviations of the eight populations of Taiwania. The locations of other mainland China populations, representing probably cultivated or naturalized trees, not used in the present study including populations Guizhou (GC), Hubei (HC), and Fujian (FC) were marked by open squares. The black circles represent locations of herbarium specimens collected from south of Gaoligonshan along Yunnan-Myanmar border (http://threatenedconifers.rbge.org.uk/taxa/details/taiwania-cryptomerioides). The population codes on the map followed Chou et al. (2011).

In the present study, AFLP and MSAP variations were surveyed in the main extant Taiwania lineages, to investigate the contrasting driving forces (i.e., drift and selection) potentially shaping population and lineage divergence and its association with specific environmental variables. Additionally, AFLP and MSAP may have the advantage of revealing genetic and epigenetic variations that could also be useful for investigating population and lineage divergence and their association with specific environmental variables. Previous studies have indicated a high rate of gene flow among mainland China populations and also between the Chinese and the Vietnamese lineages (Chou et al., 2011). A high rate of gene flow between Taiwanese populations may also be inferred as only two cpDNA haplotypes were found and one of these was restricted to a single individual within the southeastern population of Guanshan (*n* = 12, Figure 1). High rate of gene flow among populations of cypress species can be attributed to effective pollen flow due to wind-pollination as cpDNA is paternally inherited (Neale et al., 1991; Hipkins et al., 1994). Drift-mediated evolutionary processes might have played an important role in Taiwania population and lineage divergence due to high rate of gene flow. However, limited or essentially no gene flow between geographically isolated island and mainland Asia Taiwania lineages has also been indicated in Chou et al. (2011). Since the island lineage is long-diverged from mainland Asia lineages, alternative genetic and epigenetic alleles may have accumulated largely through neutral drift (Wright, 1931; Govindaraju, 1989). To test the drift divergence hypothesis, the three Taiwania lineages were investigated for the relative strength of nonadaptive and adaptive force shaping population and lineage divergence. The specific goals of this study were to: (1) characterize the gene (genetic and epigenetic) pool structure of geographically isolated Taiwania lineages; (2) test the prediction that neutral drift was the main evolutionary process driving differentiation between populations within Taiwan and between the three previously identified lineages; and (3) test the associations of environmental variables with genetic and epigenetic variations between populations and lineages.

## Materials and methods

### Sampling

Three lineages of Taiwania located in Taiwan, Yunnan-Myanmar border, and northern Vietnam were used in the present study (Table 1, Figure 1.) Populations in Taiwan are distributed at elevations of 1,800–2,600 m along the central mountain range while those in the Gaoligon mountain (Gaoligonshan) of mainland China along the Yunnan-Myanmar border occur at elevations of 1,800–2,500 m. Samples of the Gaoligonshan population included individuals collected from two stands 5 km apart. Locations of herbarium specimens (http://threatenedconifers.rbge.org.uk/taxa/details/taiwania-cryptomerioides) collected from south of Gaoligonshan, though not used in the present study, were marked by black circles in Figure 1. In northern Vietnam a single population occurs in the Hoanglien mountain (Hoanglienshan) at elevations of 1,800–2,200. We did not include samples from three other Chinese populations (populations located in Guizhou, Hubei, and Fujian provinces) in the present study because they are probably cultivated or naturalized trees. Moreover, the only cpDNA haplotype found for those populations was the most common haplotype found in the Yunnan-Myanmar lineage and the Vietnamese lineage (Chou et al., 2011). Leaf samples for DNA extraction were collected, dried in silica gel, from separate individuals in a total of eight populations: six in Taiwan (*n* = 51), one along the Yunnan-Myanmar border (mainland China) (*n* = 18), and one in northern Vietnam (*n* = 33) (Doyle and Doyle, 1987; Table 1, Figure 1). We labeled the populations with the same codes as used in Chou et al. (2011).

**Table 1:** Site properties of sampled *Taiwania* populations including the number of private allele for genetic and epigenetic markers.

**Locality**	**Region**	**Latitude Longitude**	**Altitude (m)**	***N***	**Number of private allele (*****N***_**p**_**)**		
					**AFLP**	**MSAP-m**	**MSAP-u**
Dajian (DJ)	Taiwan	24°13′37″N 121°18′23″E	2,200	8	59	7	7
Dasyueshan (DS)	Taiwan	24°15′46″N 121°00′37″E	2,400	10	56	4	11
Guanshan (GS)	Taiwan	23°12′40″N 121°04′44″E	2,400	12	56	6	14
Liwusi (LW)	Taiwan	24°06′50″N 121°32′08″E	2,200	4	31	5	15
Siouluan (SL)	Taiwan	24°33′41″N 121°16′42″E	2,100	7	18	2	10
Wangsiang (WS)	Taiwan	23°36′30″N 120°55′50″E	1,860	10	70	0	3
Gaoligongshan (YC)	China	27°42′48″N 98°34′18″E	2,300	18	4	13	12
Hoanglienshan (LV)	Vietnam	21°56′04″N 104°19′11″E	1,900	33	14	0	14
Taiwan total					290	24	60
Taiwan average					48.3 (9.61)	4 (2.61)	10 (4.47)

*Samples collected from Gaoligonshan (locality YC) represent individuals collected from two stands 5 km apart*.

*N, Number of samples; Np, number of private allele*.

*Numbers in parentheses are the standard deviation for average values of the number of private allele*.

### AFLP and MSAP

We surveyed genetic and epigenetic variation using AFLP (Vos et al., 1995) with modifications in performing ligation, pre- and selective amplification (Huang et al., 2015b) and MSAP (Xiong et al., 1999), respectively. Eleven *Eco*RI-*Mse*I selective primer combinations with sequences of the five or three bases additional to the E00 (5′-GACTGCGTACCAATTC-3′) and M00 (5′-GATGAGTCCTGAGTAA-3′) primers were used in AFLP amplification procedure (Supplementary Table 1). Eight primer combinations, were applied in selective amplification for MSAP, with additional five and two selective nucleotides, respectively, added to the E00 and HM00 (*Hpa*II-*Msp*I, 5′-ATCATGAGTCCTGCTCGG-3′) (Supplementary Table 1). AFLP and MSAP fragments were scored for each individual in the range of 100–500 bp with the software GeneMapper v.3.7 (Applied Biosystems, Foster City, CA, USA). The relative fluorescent unit threshold was set at 100. Markers with low peaks and markers separated by less than one nucleotide in a ±0.9 base pair window were removed. MSAP markers were further scored using the “mixed scoring 1” of the *MSAP-calc* R script (Schulz et al., 2013) in the R environment (R Development Core Team, 2017) and transformed to MSAP-m and MSAP-u datasets, where the last letter m and u represents methylated and unmethylated scoring types of MSAP markers, respectively. AFLP, MSAP-m, and MSAP-u datasets were deposited as Data Sheets 1–3.

Genotyping error rate (Bonin et al., 2004) per AFLP and MSAP locus was calculated as the ratio of mismatches in three amplification replicates of three randomly selected samples in each population for each primer combination. In MSAP, genotyping error rate of *Eco*RI-*Msp*I (e*Msp*I), *Eco*RI-*Hpa*II (eHpaII), and a combined error rate (e*Msp*I + e*Hpa*II – 2e_Msp*IeHpaII*_) for each primer combination were calculated (Herrera and Bazaga, 2010). The mean error rate was 7.31 and 4.85%, respectively, for AFLP and MSAP (Supplementary Table 1).

### Genetic and epigenetic diversity

AFLP, MSAP-m, and MSAP-u scored markers were used for calculation of the percentage of polymorphic loci (%*P*) at the 95% level and unbiased expected heterozygosity (*uH*_E_) (Nei, 1987) within a population using AFLP-SURV v.1.0 (Vekemans et al., 2002). Allele frequencies were estimated using the method of Zhivotovsky (1999) assuming Hardy-Weinberg equilibrium with non-uniform prior distribution. ARLEQUIN (Excoffier and Lischer, 2010) was used to calculate *uH*_E_ per locus. Nei's genetic distance (Nei, 1978) matrices of AFLP, MSAP-m, and MSAP-u datasets were calculated with the *nei.dist* function of R package poppr (Kamvar et al., 2015). Mantel correlations between the three datasets were assessed using the *mantel* function of R package vegan based on Spearman's rank correlation, and significance determined by 999 permutations.

The number of private allele for each population was calculated using the *private_alleles* function of R package poppr. Multilocus linkage disequilibrium (LD) was assessed with index of association (*I*_A_) (Brown et al., 1980) and modified index of association (*r*D) (Agapow and Burt, 2001), and calculated using the *ia* function of R poppr package. Significant departure from zero of *I*_A_ and *r*D was tested with 999 permutations.

Linear mixed effect model (LMM) with reduced maximum likelihood estimation was used to assess whether mean *uH*_E_ per locus was significantly different between populations and between lineages of Taiwania using the *lmer* function of R package lme4 (Bates et al., 2015). In LMMs, lineage and population were treated as fixed effects and locus as a random effect. Type II Wald χ^2^ test of the *Anova* function of R package car (Fox and Weisberg, 2011) was used to test the overall difference between lineages and between populations. Tukey's honest significance test for multiple comparisons was assessed using the *lsmeans* function of R package lsmeans (Lenth, 2016).

### Differentiation, relationships, and clustering

AFLP, MSAP-m, and MSAP-u datasets were used for computation of the levels of genetic and epigenetic differentiation via analysis of molecular variance (AMOVA), across population *F*_ST_, and Bayesian HICKORY θ^II^ (Holsinger and Lewis, 2003). AMOVA between populations and between lineages was computed using the *poppr.amova* function of R package poppr, and significance tested using the *randtest* function of R package ade4 with 9,999 permutations (Dray and Dufour, 2007). Across population *F*_ST_ was calculated using AFLP-SURV with 9,999 permutations. Pairwise population *F*_ST_ was calculated using ARLEQUIN with 10,000 permutations. The levels of genetic and epigenetic differentiation estimated based on θ^II^ was performed using HICKORY v.1.1 (Holsinger and Lewis, 2003). HICKORY θ^II^ is an estimate analogous to *F*_ST_, while accounting for the uncertainty associate with the inbreeding coefficient (*f*) for dominant markers. The default settings for sampling and chain length parameters (burnin = 5,000, samples = 100,000; thinning = 20) were used in HICKORY. Four models, including a full model, *f* = 0 model, θ^II^ = 0 model, and *f*-free model, were assessed for the best model fitting to the genetic and epigenetic data. The *f*-free model was used to estimate *f*. Separate analyses of the population structure, including between populations and between lineages, were performed in HICKORY. The Deviance Information Criterion (DIC) was used to identify the best fitting model. A lower D + pD (model fit + model complexity) was used to assist determination of the best model if the difference between models was smaller than six units. We ran HICKORY twice for each analysis in order to check the convergence of parameters.

Considering that complex evolutionary processes such as gene flow between populations and recombination within a population can lead to conflict or ambiguous phylogenetic signals in a single tree representation, Neighbor-Net analysis (Bryant and Moulton, 2004) was used to reveal Taiwania population and lineage relationships, based on Nei's genetic distance using the *neighborNet* function of R package phangorn (Schliep, 2011; Schliep et al., 2017). The bootstrap support values were calculated with the *aboot* function of R package poppr.

To identify genetically and epigenetically homogeneous groups of Taiwania populations, we used the Bayesian model-based method implemented in STRUCTURE v.2.3 (Pritchard et al., 2000; Falush et al., 2007), the sparse non-negative factorization (sNMF) method implemented in R package LEA (Frichot and Francois, 2015), and the discriminant analysis of principal components (DAPC) method implemented in R package adegenet (Jombart et al., 2010; Jombart and Ahmed, 2011). In STRUCTURE, we assumed an admixture model with an informative prior of sampling location. *K* values ranging from 1 to 9 were tested with 10 replicate runs for each *K* with 10^6^ iterations and 10^5^ burn-in steps. We used R package pophelper (Francis, 2017) to summarize the mean log probability (Ln*P*(D)) (Pritchard et al., 2000), change in the log probability (Δ*K*) (Evanno et al., 2005), and symmetric similarity coefficient (SSC) (Jakobsson and Rosenberg, 2007) for evaluation of clustering outcomes at each *K*. The *snmf* function of R package LEA was used to assess individual assignments based on sNMF algorithm with least-squares optimization. In *snmf* of LEA, regularization parameter, iterations, and repetitions were set to 100, 200, and 10, respectively, with other arguments set to defaults for *K* = 1–8. The best *K* was evaluated with the means of minimal cross-entropy (CE). DAPC, a multivariate method, was performed using the *find.clusters* and *dapc* functions of R package adegenet. DAPC first performed a principal component analysis (PCA) followed by a discriminant analysis that maximize the inter-group component of variation.

### Potential outlier detection

Genome scan methods of DFDIST within the Mcheza workbench (Antao and Beaumont, 2011) and BAYESCAN v.2.1 (Foll and Gaggiotti, 2008) were used to test for *F*_ST_ outliers in global and pairwise comparisons. In DFDIST, outliers were identified by comparing observed distribution with neutral expectations at a 99.5% confidence interval (CI) and an FDR of 1% with each run comprising 10^6^ simulations. Both “neutral mean *F*_ST_” and “force mean *F*_ST_” were selected. *F*_ST_ outliers were removed to increase the reliability of calculating the global distribution of *F*_ST_. BAYESCAN uses a reversible-jump Markov chain Monte Carlo algorithm based on a Bayesian likelihood approach to estimate the ratio of posterior probabilities of selection over neutrality [the posterior odds (PO)]. Parameters for running BAYESCAN were 100 pilot runs of 50,000 iterations followed by a sample size of 50,000 with thinning interval of 20 among 10^6^ iterations. A locus with log_10_(PO) > 0.5 was considered to have substantial evidence for selection (Jeffreys, 1961).

A total of 32 *F*_ST_ outliers were identified using DFDIST and BAYESCAN in global and pairwise lineage comparisons (see section Results). Because genome scan methods may obtain low support value [low log_10_(PO)] in BAYESCAN and false positives in DFDIST (Pérez-Figueroa et al., 2010), Samβada (Stucki et al., 2017) was further used to test whether allele frequencies of *F*_ST_ outliers identified either by DFDIST or by BAYESCAN had significant associations with the values of environmental variables using multiple univariate logistic regression based on generalized linear model (GLM). For the Samβada analysis, genetic and epigenetic markers were coded with “11” and “00” for presence and absence and tested for associations of allele frequencies with values of environmental variables (BIO4, BIO15, NDVI, PET, aspect, and slope) in global and pairwise lineage comparisons. Significant fit was identified comparing between models with and without environmental variables based on both Wald and G scores with an FDR cutoff of 0.01. When only Taiwanese populations were investigated, no *F*_ST_ outlier was found using DFDIST and BAYESCAN, and neither with Samβada for association between genetic and epigenetic variations with environmental variables. Therefore, we focused on lineage comparison in the following analyses.

Generalized linear mixed effect models (GLMMs) were also used to test for the association of *F*_ST_ outliers with environmental variables (Lobréaux and Melodelima, 2015). We performed GLMMs with a logit link function and a binomial residual distribution in analyzing the 32 *F*_ST_ outliers (response variables) using the *glmer* function of R package lme4. In GLMMs, environmental variables were used as fixed effects and lineage as a random effect. To determine significant associations of environmental gradients with allele frequencies of *F*_ST_ outliers, profile CIs (95 and 99%) based on likelihood ratio test for fixed effects were used. A two locus exact test was used to assess pairwise LD between potential outlier loci using ARLEQUIN, and significance determined by 10,000 permutations.

### Relative contribution of environment and geography in explaining genetic and epigenetic variation between taiwania lineages

Environmental variables used were classified into three categories (i.e., bioclimate, topological, and ecological variables; Supplementary Table 2). Nineteen bioclimate variables for sample sites were downloaded from the WorldClim v.1.4 (http://www.worldclim.org/) at 30-s spatial resolution (~1 km) (Hijmans et al., 2005). Topographic (aspect and slope) variables were derived from a 30-m resolution ASTER GDEM (Global Digital Elevation Map; http://lpdaac.usgs.gov). Ecological factors including normalized difference vegetation index (NDVI) and enhanced vegetation index (EVI) derived from moderate resolution imaging spectroradiometer (MODIS) dataset MOD13A2 (1 km resolution), and leaf area index (LAI) and fraction of absorbed photosynthetically active radiation (fPAR) obtained based on MOD15A2 dataset (500 m resolution). The annual total potential evapotranspiration (PET) was calculated based on MOD16A3 dataset (500 m resolution). All the MODIS datasets were acquired from Land Process Distributed Active Archive Center (LPDAAC, http://lpdaac.usgs.gov) during 2001–2013. Four tiles (H26V06, H27V06, H28V06, and H29V06) were required to cover the entire study region. The monthly mean values of NDVI, EVI, LAI, and fPAR were computed using a maximum values composite procedures (Huete et al., 2002). The land cover types of population locations were extracted from a 500 m resolution MODIS product MCD12Q1 of 2013 (Figure 1, Friedl et al., 2010). Taiwania in Taiwan and along the Yunnan-Myanmar border occur in a mixed forest land cover type while the Vietnamese lineage occurs in evergreen broadleaf forests.

Analyses that were focused only on Taiwanese populations included seven additional ecological factors: annual moisture index, relative humidity (RH), cloud cover (CLO), time of sunshine (SunH), number of rainfall days per year (RainD), mean wind speed (WSmean), and soil pH. Monthly mean values of RH, CLO, SunH, RainD, and WSmean at spatial resolution of 1 km were obtained from the Data Bank for Atmospheric Research (DBAR, https://www.narlabs.org.tw/en/, recorded in 1990–2013) using a universal spherical model of the Kriging method in ArcGIS (Chang et al., 2014). Soil pH values of sample sites, based on an island-wide soil investigation (*n* = 1150) conducted in 1969–1986, were acquired from the Agriculture and Food Agency of Taiwan (Chang et al., 2009). Annual precipitation and annual potential evapotranspiration (derived from annual mean temperature) of each sample sites were used to calculate annual moisture index (Thornthwaite, 1948).

Correlations between environmental variables and variance inflation factor (VIF) were calculated using the *cor* function of R and *vif* function of R package usdm (Naimi et al., 2014), respectively. When all environmental variables were used in VIF calculation, high collinearity among environmental variables were found (Supplementary Table 2), hence we performed VIF calculation for environmental variables within each category (i.e., bioclimatic, topographic, and ecological factors). Finally, environmental variables with VIF > 5 and which were strongly correlated with other variables (|r| > 0.8) within each environmental category were removed. Six environmental variables including BIO4 (monthly temperature variation), BIO15 (monthly precipitation variation), PET, NDVI, aspect, and slope were retained when the three Taiwania lineages were considered (Supplementary Figures 1, 2). Eight environmental variables (BIO4, BIO15, CLO, RainD, WSmean, NDVI, aspect, and slope) were retained when only Taiwanese populations were considered (Supplementary Figures 1, 2).

Permutational multivariate analysis of variance (PERMANOVA) was used to test for environmental heterogeneity among Taiwania populations within Taiwan and among Taiwania lineages using the *adonis* function of R package vegan (Oksanen et al., 2017). Euclidean distance matrices of environmental variables were generated and used as response variable for PERMANOVA with 999 permutations. Pairwise comparisons were also conducted using the *pairwise.perm.manova* function of R package RVAideMemoire (Herve, 2018) with 999 permutations and a false discovery rate (FDR) of 5%.

The six retained environmental variables, for between lineage comparisons, were used in a PCA to obtain non-redundant PCs for variation partitioning of genetic and epigenetic variations explained by environment and geography. PCA was performed using the *prcomp* function of R. The first two PCs with eigenvalues >1 (PC1: 3.098; PC2: 1.687) explaining 51.63 and 28.12% of environmental variation were used in a redundancy analysis to assess the relative contribution of environmental variables explaining the total genetic and epigenetic variations using the *varpart* function of R package vegan, and significance tested using the *anova.cca* function with 999 permutations. AFLP, MSAP-m, and MSAP-u variations were, respectively, partitioned into four fractions explained by pure environmental variables (fraction [a]), geographically-structured environmental variables (fraction [b]), pure geographic variables (fraction [c]), and residual effects (fraction [d]) (Borcard et al., 1992; Borcard and Legendre, 2002), based on adjusted *R*^2^-values (Peres-Neto et al., 2006). Longitude and latitude of sample localities were used as geographic variables in the analysis.

To identify specific environmental variables significantly explaining the 32 *F*_ST_ outliers, forward selection was performed using the *forward.sel* function of R package packfor (Dray et al., 2016). The double-stopped criterion (Blanchet et al., 2008), i.e., selection stopped if either the conventional level of significance (α < 0.05) or the global adjusted *R*^2^ was exceeded, was applied in the selection procedure to prevent overestimation of the explained variance. The three categories of environmental variables were used separately in the forward selection analysis.

To further assess the relative importance of environmental variables influencing outlier genetic and epigenetic variations, functions within the R package MuMIn (Barton, 2018) were used. GLMM models mentioned above were used for the *dredge* function that fits all possible models for each outlier (response variable) and performed the subsequent model averaging analyses based on Akaike information criterion with a correction for small sample sizes (AICc) (ΔAICc ≤ 3, the *model.avg* function). AICc was used to rank the models and to calculate the Akaike weights for each model (Burnham and Anderson, 2002). A 95% confidence set of models was determined for each analysis performed and used to determine 95% CIs containing the best-approximating model to the best model conditioned on all parsimonious (ΔAICc ≤ 3) models. The relative importance of environmental variables contribution to explaining variations of outlier genetic and epigenetic loci was assessed using the *importance* function. The 95% CIs did not bracket zero were used to provide evidence for an association between the most important environmental variable (predictor) and the presence of a genetic or epigenetic outlier (response variable), and a marginal-*R*^2^ for the fixed effect of the most important environmental variable(s) explaining outlier variation was calculated using the *r.squaredGLMM* function.

## Results

### Correlation between genetic and epigenetic variation

In total, 1,413 and 462 loci were obtained, respectively, with a mean ± SD of 128.45 ± 10.46 and 57.75 ± 9.85, for AFLP and MSAP (Supplementary Table 1). We obtained 456 MSAP-m and 289 MSAP-u markers for the 462 MSAP loci. Mantel tests revealed significant correlations between genetic and epigenetic variations and also between the two types of epigenetic variation (AFLP vs. MSAP-m, Mantel *r* = 0.387, *P* < 0.001; AFLP vs. MSAP-u, Mantel *r* = 0.388, *P* < 0.001; and MSAP-m vs. MSAP-u, Mantel *r* = 0.680, *P* < 0.001).

### Diversity, differentiation, and inbreeding

The number of private AFLP alleles was comparatively higher in populations of the Taiwanese lineage (*N*_p_ ranged from 18 to 70; mean ± SD: 48.3 ± 19.60) than those of Yunnan-Myanmar (*N*_p_ = 4) and Vietnamese (*N*_p_ = 14) lineages (Table 1). A comparatively higher number of MSAP-m private alleles were found in the Yunnan-Myanmar lineage (*N*_p_ = 13) compared with Vietnamese (*N*_p_ = 0) and Taiwanese (*N*_p_ ranged from 0 to 7, mean ± SD: 4 ± 2.61) lineages at the population level. The Yunnan-Myanmar and Vietnamese lineages had similar number of MSAP-u private alleles (*N*_p_ = 12 and 14, respectively) compared to populations of Taiwanese lineage (*N*_p_ ranged from 3 to 15, mean ± SD: 10 ± 4.47). The average percentage of polymorphism was comparatively higher in Taiwanese lineage compared with Yunnan-Myanmar and Vietnamese lineages (AFLP, MSAP-m, and MSAP-u, respectively, were 66.1, 59.0, and 49.3% in Taiwanese; 37.2, 49.6, 43.3% in Yunnan-Myanmar; and 37.4, 41.9, and 33.2% in Vietnamese lineages; Table 2). The %*P* and *uH*_E_ indices were not positively dependent on population sample size based on Spearman's rank correlation test (%*P*: AFLP, ρ = −0.228, *P* = 0.588; MSAP-m, ρ = −0.084, *P* = 0.844; MSAP-u, ρ = −0.754, *P* = 0.031; *uH*_E_: AFLP, ρ = −0.753, *P* = 0.031; MSAP-m, ρ = −0.850, *P* = 0.007; MSAP-u, ρ = −0.539, *P* = 0.168).

**Table 2:** Population genetic and epigenetic parameters of the eight populations of Taiwania.

**Population**	**AFLP**				**MSAP-m**				**MSAP-u**			
	**%*P***	***uH*_E_** ** (SE)**	***I*_A_** ** (*P*)**	***r*D** ** (*P*)**	**%*P***	***uH*_E_** ** (SE)**	***I*_A_** ** (*P*)**	***r*D** ** (*P*)**	**%*P***	***uH*_E_** ** (SE)**	***I*_A_** ** (*P*)**	***r*D** ** (*P*)**
DJ	67.7	0.249 (0.005)	31.97 (0.001)	0.0348 (0.001)	61.6	0.158 (0.005)	3.97 (0.001)	0.0143 (0.001)	51.2	0.157 (0.010)	5.33 (0.001)	0.0382 (0.001)
DS	66.9	0.217 (0.005)	22.48 (0.001)	0.0247 (0.001)	61.0	0.150 (0.006)	1.35 (0.001)	0.0050 (0.001)	55.4	0.167 (0.010)	4.03 (0.001)	0.0287 (0.001)
GS	72.3	0.239 (0.005)	27.45 (0.001)	0.0279 (0.001)	69.1	0.147 (0.005)	2.19 (0.001)	0.0071 (0.001)	33.6	0.129 (0.009)	6.38 (0.001)	0.0416 (0.001)
LW	56.4	0.254 (0.005)	16.17 (0.001)	0.0223 (0.001)	41.2	0.161 (0.007)	1.34 (0.001)	0.0073 (0.047)	56.7	0.253 (0.011)	3.75 (0.001)	0.0293 (0.001)
SL	59.7	0.217 (0.005)	20.46 (0.001)	0.3744 (0.001)	62.7	0.171 (0.005)	1.50 (0.001)	0.0053 (0.004)	43.9	0.137 (0.009)	3.19 (0.001)	0.0262 (0.001)
WS	73.5	0.242 (0.005)	30.21 (0.001)	0.0492 (0.001)	58.6	0.142 (0.006)	0.22 (0.001)	0.0001 (0.203)	55.0	0.168 (0.010)	2.38 (0.001)	0.0167 (0.001)
YC	37.2	0.125 (0.005)	18.85 (0.001)	0.0194 (0.001)	49.6	0.149 (0.007)	0.794 (0.001)	0.0029 (0.001)	43.3	0.162 (0.011)	1.55 (0.001)	0.0103 (0.001)
LV	37.4	0.144 (0.005)	3.94 (0.001)	0.0086 (0.001)	41.9	0.128 (0.006)	0.80 (0.001)	0.0028 (0.001)	33.2	0.123 (0.010)	0.76 (0.001)	0.0054 (0.001)
Taiwan average	66.1	0.236			59.0	0.155			49.3	0.169		
Total average	58.9	0.211			55.7	0.151			46.5	0.162		

*%P, the percentage of polymorphic loci; uH_E_ is the biased expected heterozygosity. I_A_, index of association; rD, modified index of association*.

The levels of *uH*_E_ in Taiwanese populations ranged between 0.217 and 0.254 for AFLP, between 0.142 and 0.171 for MSAP-m, and between 0.129 and 0.253 for MSAP-u (Table 2). The levels of *uH*_E_ of Yunnan-Myanmar lineage were 0.125, 0.149, and 0.162, respectively, for AFLP, MSAP-m, and MSAP-u. The levels of *uH*_E_ in the Vietnamese lineage were 0.144, 0.128, and 0.123, respectively, for AFLP, MSAP-m, and MSAP-u. Significant differences were found between Taiwania lineages (LMMs; AFLP: χ^2^ = 1350.3, *P* < 0.0001; MSAP-m: χ^2^ = 49.76, *P* < 0.0001; MSAP-u: χ^2^ = 51.17, *P* < 0.0001). *Post-hoc* pairwise comparisons revealed significantly higher levels of AFLP, MSAP-m, and MSAP-u diversity in the Taiwanese lineage compared with the other two lineages, and significant differences in these diversity measures between the Yunnan-Myanmar and the Vietnamese lineages were also found (Supplementary Table 4). Moreover, the levels of genetic and epigenetic diversity were significantly different overall among Taiwania populations occurring in Taiwan (AFLP: χ^2^ = 215.69, *P* < 0.0001; MSAP-m: χ^2^ = 19.79, *P* = 0.0014; MSAP-u: χ^2^ = 43.38, *P* < 0.0001). However, not all pairwise population comparisons showed significant differences in the level of *uH*_E_ (Supplementary Table 4).

AMOVA, *F*_ST_, and HICKORY θ^II^ revealed consistent results in the levels of differentiation between lineages and between populations in all three datasets (AFLP, MSAP-m, and MSAP-u) analyzed using the total data (Table 3). The HICKORY results suggest that the full models fitted best to the data (AFLP, MSAP-m, and MSAP-u) based on DIC and D + pD values (Supplementary Table 5). We found moderate but significant levels of differentiation for AFLP and MSAP-u comparing between lineages and between populations according to AMOVA and θ^II^ (Table 3). However, low levels of differentiation, albeit significant, were found for MSAP-m, between lineages and between populations. Across lineage and across population *F*_ST_ showed similar trends of the level of differentiation as revealed in AMOVA and θ^II^, however, with lower values. High levels of significant pairwise *F*_ST_ were found when compared between populations of Taiwanese lineage and populations of Yunnan-Myanmar and Vietnamese lineages (Supplementary Table 6). However, non-significant levels of pairwise population *F*_ST_ were found when only Taiwanese populations were compared.

**Table 3:** Summary of the analysis of molecular variance (AMOVA), *F*_ST_, and θ^II^.

**Source of variation**	**Genetic differentiation**
**AFLP**	
Between lineages	Φ_CT_ = 0.1503 (<0.001) *F*_ST_ = 0.0978 (<0.001)
	θ^II^ = 0.135 (0.125, 0.146)
Between populations	Φ_ST_ = 0.1516 (<0.001) *F*_ST_ = 0.0783 (<0.001)
	θ^II^ = 0.125 (0.119, 0.132)
**MSAP-m**	
Between lineages	Φ_CT_ = 0.0572 (<0.001) *F*_ST_ = 0.0313 (<0.001)
	θ^II^ = 0.047 (0.030, 0.057)
Between populations	Φ_ST_ = 0.0588 (<0.001) *F*_ST_ = 0.0134 (<0.001)
	θ^II^ = 0.053 (0.033, 0.064)
**MSAP-u**	
Between lineages	Φ_CT_ = 0.1608 (<0.001) *F*_ST_ = 0.1062 (<0.001)
	θ^II^ = 0.135 (0.134, 0159)
Between populations	Φ_ST_ = 0.1785 (<0.001) *F*_ST_ = 0.0947 (<0.001)
	θ^II^ = 0.148 (0.131, 0.165)

Results represent comparison between the three lineages and eight populations of Taiwania sampled in this study.

*Values within parentheses are P values for AMOVA and F_ST_ and 95% confidence intervals for θ^II^*.

HICKORY was also used to assess the contemporary reproductive mode of Taiwania using the *f*-free model and results gave an estimate of *f* around 0.5 analyzed either at lineage or population level in all three datasets (Supplementary Table 5). *I*_A_ and *r*D, the measures of multilocus LD, showed significant values departure from random association between loci of AFLP, of MSAP-m, and of MSAP-u (Table 2).

### Population and lineage clustering

**Figure 2:**
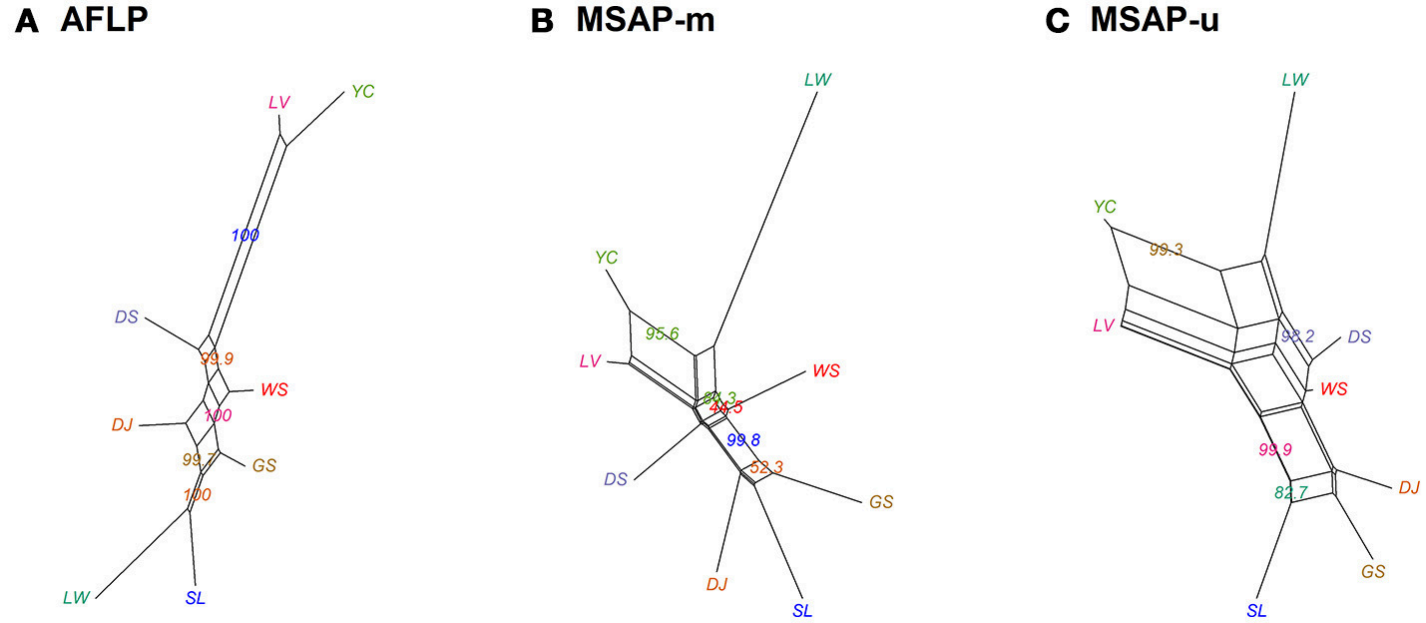
Neighbor-Net graph for the eight populations of Taiwania based on Nei's genetic distance, with bootstrap support values displayed. **(A)** AFLP, **(B)** MSAP-m, and **(C)** MSAP-u. See Table 1 for abbreviations of the eight populations of Taiwania.

**Figure 3:**
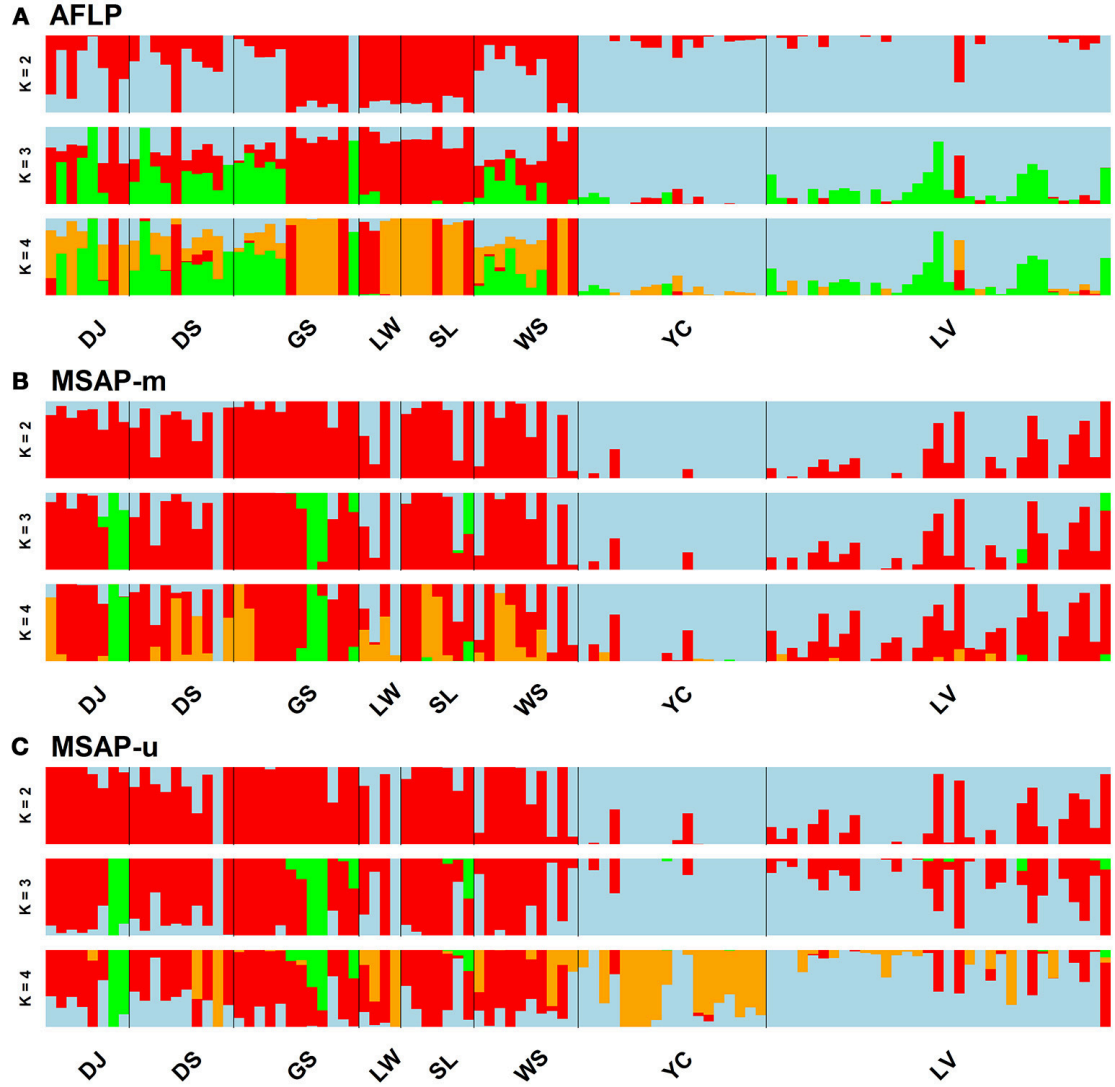
Individual assignments analyzed using LEA for clustering scenarios of *K* = 2–4. The subpanels display results analyzed based on the **(A)** AFLP, **(B)** MSAP-m, and **(C)** MSAP-u datasets, respectively. See Table 1 for abbreviations of the eight populations of Taiwania.

**Figure 4:**
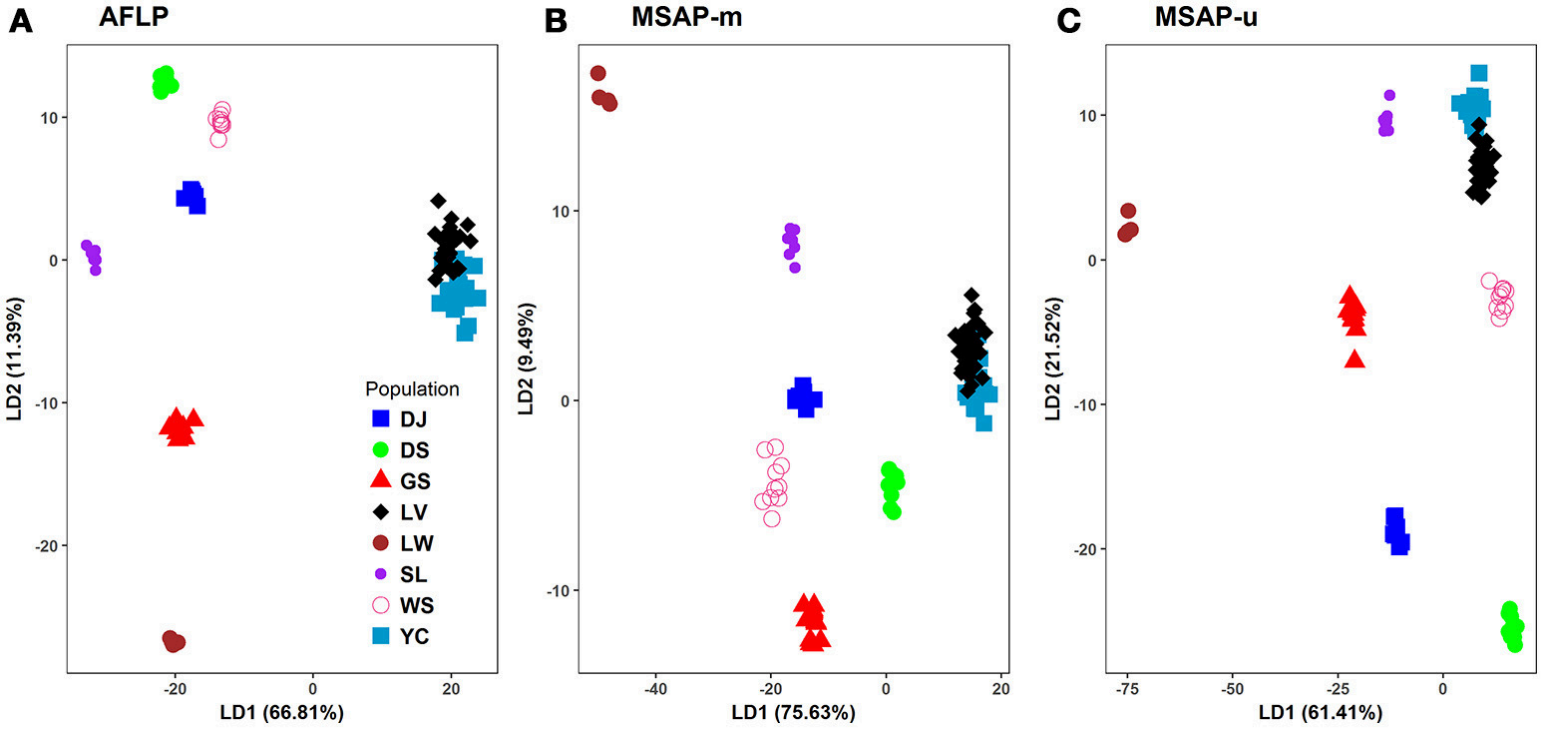
Scatter plots of the first two linear discriminants analyzed using discriminant analysis of principal components (DAPC). **(A)** AFLP, **(B)** MSAP-m, and **(C)** MSAP-u. See Table 1 for abbreviations of the eight populations of Taiwania.

Population and lineage relationships were assessed using Neighbor-Net analysis (Figure 2). Genetic and epigenetic homogeneous groups of individuals were assessed using STRUCTURE, LEA, and DAPC (Supplementary Figure 4, Figures 3, 4). Neighbor-Net analysis consistently revealing the close relationship between Yunnan-Myanmar and Vietnamese lineages differentiated from Taiwanese populations based on AFLP, MSAP-m, and MSAP-u datasets (Figure 2). In the STRUCTURE analysis, the highest Ln*P*(D) was, respectively, obtained at *K* = 6, 3, and 7, for AFLP, MSAP-m, and MSAP-u (Supplementary Figure 3A). However, the maximal Δ*K* occurred at *K* = 2 for all three datasets (Supplementary Figure 3B). SSC values were high (>0.997) for *K* = 2–3 in all three datasets and dropped when *K* = 4, particularly for MSAP-m and MSAP-u, but fluctuations in SSC values were observed at *K* = 5–9 (Supplementary Figure 3C). In the LEA analysis, CE was minimized at *K* = 4, 2, and 5, respectively, for AFLP, MSAP-m, and MSAP-u (Supplementary Figure 3D). LEA and STRUCTURE clustering results were depicted for *K* = 2–4 (Figure 3, Supplementary Figure 4, respectively) because *K* > 4 revealed no further information regarding to individual assignments for Taiwania samples examined. LEA and STRUCTURE analyses revealed a clear phylogeographic break distinguishing Taiwanese populations from Yunnan-Myanmar and Vietnamese lineages when *K* = 2 (island cluster: populations of Taiwanese lineage; mainland Asia cluster: populations of Yunnan-Myanmar and Vietnamese lineages). When *K* = 3 and 4, no further differentiation power between lineages was found based on AFLP and MSAP-m data. Nonetheless, LEA for MSAP-u showed differentiation between Yunnan-Myanmar and Vietnamese lineages at *K* = 4 (Figure 3).

In DAPC analysis, 90% of PCA variance in both genetic and epigenetic data was used in discriminant analysis. The PCA eigenvalues for the first two PCs were 5.28 and 2.32, 0.73 and 0.36, and 1.02 and 0.55, respectively for AFLP, MSAP-m, and MSAP-u. The eigenvalues for the first two DAPC linear discriminants were 6,141.35 and 1047.21, 4,364.64 and 547.77, and 5504.83 and 1929.34, respectively, for AFLP, MSAP-m, and MSAP-u. The amounts of genetic and epigenetic variation explained by the first two linear discriminants were, 66.81 and 11.39%, 75.63 and 9.49%, and 61.41 and 21.52%, respectively, for AFLP, MSAP-m, and MSAP-u (Figure 4). DAPC results also displayed close relationship between Yunnan-Myanmar and Vietnamese lineages in agreement with the Neighbor-Net, LEA, and STRUCTUTE results. However, varied phylogeographic relationships between Taiwanese populations were observed in DAPC in the AFLP, MSAP-m, and MSAP-u datasets. Nonetheless, DAPC also depicted clear differentiation of Taiwanese individuals from those of Yunnan-Myanmar and Vietnamese lineages genetically and epigenetically.

### Partitioning genetic and epigenetic variation explained by environment and geography

Overall significant environmental heterogeneity, based on the six retained environmental variables (Supplementary Table 2), was found between the three Taiwania lineages using PERMANOVA (*P* = 0.001). Environmental heterogeneity was also found in pairwise lineage comparisons (all *P*s = 0.001). Moreover, PERMANOVA revealed overall environmental heterogeneity based on the eight retained environmental variables when only the six populations of Taiwanese lineage were analyzed (*P* = 0.001). However, not all pairwise population comparisons showed significant environmental differences (Supplementary Table 3).

The six retained environmental variables, for the three Taiwania lineages, were analyzed with PCA. The first PC was used to assess the amounts of genetic and epigenetic information explained purely by environmental variables using variation partitioning. Because no significant amount of either genetic or epigenetic variation was found to be explained by the second environmental PC, we show here only the results analyzed for environmental PC1 (Table 4). Large amounts of genetic and epigenetic variations were unaccounted for in all three datasets. The total amount of variation explained by both environment and geography were, 11.23, 3.57, and 11.09%, respectively, for AFLP, MSAP-m, and MSAP-u. The portion of variation explained by pure geography independent of any environmental variables measured was larger compared to the portion explained by non-geographically structured environmental variables in all three datasets. Geography explained significant amount of genetic and epigenetic variations in all three datasets. Nonetheless, significant pure environmental effects, albeit small, were also found for AFLP and MSAP-u.

**Table 4:** The percentage of variation explained in genetic and epigenetic loci accounted for by non-geographically-structured environmental variables [a], shared (geographically-structured) environmental variables [b], pure geographic factors [c], and undetermined component [d] analyzed based on environmental PC1.

	**Variation (adjusted *R*^2^)**	***F***	***P***
**AFLP**			
Environment [a]	0.01243	2.3862	0.001
Environment + Geography [b]	0.04759	–	–
Geography [c]	0.05226	3.9433	0.001
[a + b + c]	0.11227	5.2579	0.001
Residuals [d]	0.88773	–	–
**MSAP-m**			
Environment [a]	0.00029	1.0301	0.358
Environment + Geography [b]	0.01680	–	–
Geography [c]	0.01857	1.9629	0.001
[a + b + c]	0.03567	2.2453	0.001
Residuals [d]	0.96433	–	–
**MSAP-u**			
Environment [a]	0.00452	1.5032	0.015
Environment + Geography [b]	0.05451	–	–
Geography [c]	0.05187	3.9168	0.001
[a + b + c]	0.11089	5.199	0.001
Residuals [d]	0.88911	–	–

*Proportions of explained variation were obtained from variation partitioning by redundant analysis. F and P values are specified for testable fractions. Fraction [b] is untestable because the adjusted R^2^ value was obtained by subtraction ([a + b + c]-[a]-[c]) rather than by estimation*.

### Potential outliers under selection and the most important environmental factors explaining genetic and epigenetic variations

In total, 32 loci (15 AFLP, 1.06%; 4 MSAP-m, 0.88%; and 13 MSAP-u, 4.50%) were detected as potential outliers under selection based either on DFDIST or BAYESCAN (Table 5). Multiple univariate logistic regression using Samβada found these 32 potential outliers associated strongly with environmental variables in either global or pairwise lineage comparisons (Table 5). GLMM results showed 13 of 15 AFLP, 3 of 4 MASP-m, and 10 of 13 MSAP-u outliers were strongly associated with environmental variables either at 95 or 99% CIs (Table 5). Unexpectedly, neither genome scan methods (DFDIST and BAYESCAN) nor correlation based method (Samβada) found potential selective outliers for Taiwania populations within Taiwan in global and pairwise population comparisons, albeit PERMANOVA revealed significant environmental heterogeneity among populations.

**Table 5:** Summary of outliers potentially evolved under selection identified by frequency based genome scan methods (DFDIST and BAYESCAN), generalized linear model (Samβada), and generalized linear mixed effect model (GLMM) based on AFLP, MSAP-m, MSAP-u datasets.

**Marker**	***F***_**ST**_ **based method**		**Sam**βada^*^and generalized linear mixed effect model (95% CI^**^, 99% CI^***^)					
	**DFDIST (*P* value)**	**BAYESACN log_10_(PO)**	**BIO4**	**BIO15**	**NDVI**	**PET**	**Aspect**	**Slope**
**AFLP**
aP1_264	0.00030^a^ 0.00001^b^^c^	2.4186^a^ 0.6498^b^	^*^^a^, ^b^, ^c^	^*^^a^, ^b^, ^c^	^*^^a^, ^b^, ^c^			^*^^a^, ^c^
aP1_377	0.00033^a^ 0.00001^b^		^*^^a^, ^b^	^*^^a^, ^b^	^*^^b^		^*^^a^, ^b^	
aP2_195	0.00001^b^		^*^^b^ ^**^ ^***^		^*^^b^ ^**^	^**^ ^***^	^**^ ^***^	^**^ ^***^
aP2_204	0.00001^b^		^*^^b^ ^**^ ^***^	^**^	^*^^b^ ^**^	^**^ ^***^	^*^^b^ ^**^ ^***^	^**^ ^***^
aP4_287	0.00001^b^		^*^^b^ ^**^ ^***^		^**^	^**^ ^***^	^**^ ^***^	^**^ ^***^
aP5_139		0.7426^a^	^*^^a^ ^**^ ^***^	^*^^a^ ^**^	^*^^a^ ^**^ ^***^	^**^ ^***^	^**^ ^***^	^**^ ^***^
aP5_168	0.00001^b^		^*^^b^ ^**^ ^***^		^*^^b^		^*^^b^	^**^
aP9_133	0.00013^b^		^*^^b^	^*^^b^ ^**^ ^***^	^*^^b^		^*^^b^ ^**^ ^***^	^**^
aP9_322		0.6276^a^	^*^^a^ ^**^ ^***^	^*^^a^ ^**^ ^***^	^*^^a^ ^**^ ^***^	^**^ ^***^	^**^ ^***^	^**^ ^***^
aP9_391		1.0651^a^	^*^^a^	^*^^a^	^*^^a^			
aP12_243	0.00001^d^		^*^^d^	^*^^d^ ^**^ ^***^	^*^^d^	^*^^d^ ^**^	^*^^d^ ^**^	^*^^d^ ^**^ ^***^
aP13_142	0.00515^b^				^*^^b^ ^**^ ^***^	^**^ ^***^		^**^ ^***^
aP13_160		1.5681^a^	^*^^a^	^*^^a^ ^**^ ^***^	^*^^a^			^*^^a^
aP13_235		0.8470^a^	^*^^a^	^*^^a^	^*^^a^			
aP13_285		1.2573^a^	^*^^a^ ^**^ ^***^	^*^^a^ ^**^ ^***^	^*^^a^			^*^^a^ ^**^
**MSAP-m**
mP7MH_201	0.00001^b^		^*^^b^ ^**^		^*^^b^ ^**^ ^***^			
mP9MH_207	0.00008^a^ 0.00001^b^	1.5157^a^ 0.8856^b^	^*^^a^, ^b^ ^**^ ^***^	^**^ ^***^	^*^^b^			
mP9MH_214	0.00001^a^ 0.00001^b^	1.3445^a^ 0.8623^b^	^*^^a^		^*^^b^			
mP16MH_198	0.00013^a^ 0.00001^b^	1.2910^a^ 0.5036^b^	^*^^a^, ^b^		^*^^b^ ^**^ ^***^			
**MSAP-u**
uP5MH_169	0.00001^b^		^*^^b^ ^**^ ^***^	^**^ ^***^	^*^^b^	^**^ ^***^	^*^^b^ ^**^ ^***^	^**^ ^***^
uP6MH_135		0.5684^a^	^*^^a^ ^**^ ^***^	^*^^a^ ^**^ ^***^	^*^^a^	^*^^a^ ^**^ ^***^	^**^ ^***^	^*^^a^ ^**^ ^***^
uP9MH_158	0.00001^b^		^*^^b^ ^**^		^*^^b^ ^**^ ^***^			
uP13MH_117	0.00001^b^		^*^^b^ ^**^ ^***^	^**^ ^***^	^*^^b^ ^**^ ^***^	^**^ ^***^	^*^^b^ ^**^ ^***^	^**^ ^***^
uP14MH_102	0.00057^c^		^*^^c^	^*^^c^	^**^ ^***^			^*^^c^
uP14MH_209	0.00001^b^		^*^^b^ ^**^ ^***^	^*^^b^	^*^^b^	^**^ ^***^	^*^^b^ ^**^ ^***^	^**^ ^***^
uP14MH_255	0.00001^b^		^*^^b^		^*^^b^ ^**^ ^***^			
uP15MH_106	0.00320^d^		^*^^d^ ^**^ ^***^	^*^^d^ ^**^ ^***^	^*^^d^ ^**^ ^***^	^*^^d^ ^**^ ^***^	^*^^d^ ^**^ ^***^	^*^^d^ ^**^ ^***^
uP15MH_134	0.00001^b^		^*^^b^ ^**^ ^***^	^*^^b^	^*^^b^		^*^^b^	
uP15MH_227	0.00001^b^^c^	0.6766^a^	^*^^a^, ^b^, ^c^	^*^^a^, ^b^, ^c^	^*^^a^, ^c^		^*^^b^	^*^^c^
uP16MH_169	0.00001^b^		^*^^b^ ^**^ ^***^	^*^^b^	^*^^b^		^*^^b^ ^**^ ^***^	
uP16MH_248	0.00001^b^		^*^^b^					
uP16MH_339	0.00001^b^		^*^^b^					

In GLMM, only the 32 potential selective outliers identified either by DFDIST or BAYESCAN were analyzed.

* *P < 0.0001 after 1% FDR cut off (both Wald and G tests)in Samβada analysis*.

** Values do not bracket zero in 95% confidence intervals in GLMM.

*** *Values do not bracket zero in 99% confidence intervals in GLMM*.

a Global comparison among three Taiwania lineages.

b Pairwise comparison between Taiwanese and Yunnan-Myanmar lineages.

c Pairwise comparison between Taiwanese and Vietnamese lineages.

d *Pairwise comparison between Yunnan-Myanmar and Vietnamese lineages*.

In forward selection, environmental variables were classified into three categories (i.e., bioclimate, ecology, and topology) considering the redundancy between variables in different categories in explaining genetic and epigenetic variations and also because of only small amounts of genetic and epigenetic variations attributed to pure environment in the variation partitioning analysis (Table 4). For all three datasets, forward selection showed that monthly temperature variation, NDVI, and aspect, respectively, was the most important bioclimatic, ecological, and topological factor explaining variation (Supplementary Table 7). Forward selection consistently found that environmental variables including monthly temperature variation, NDVI, and aspect significantly explained outlier genetic and epigenetic variations between Taiwania lineages.

### Relative importance of environmental variables explaining potential selective outliers

**Figure 5:**
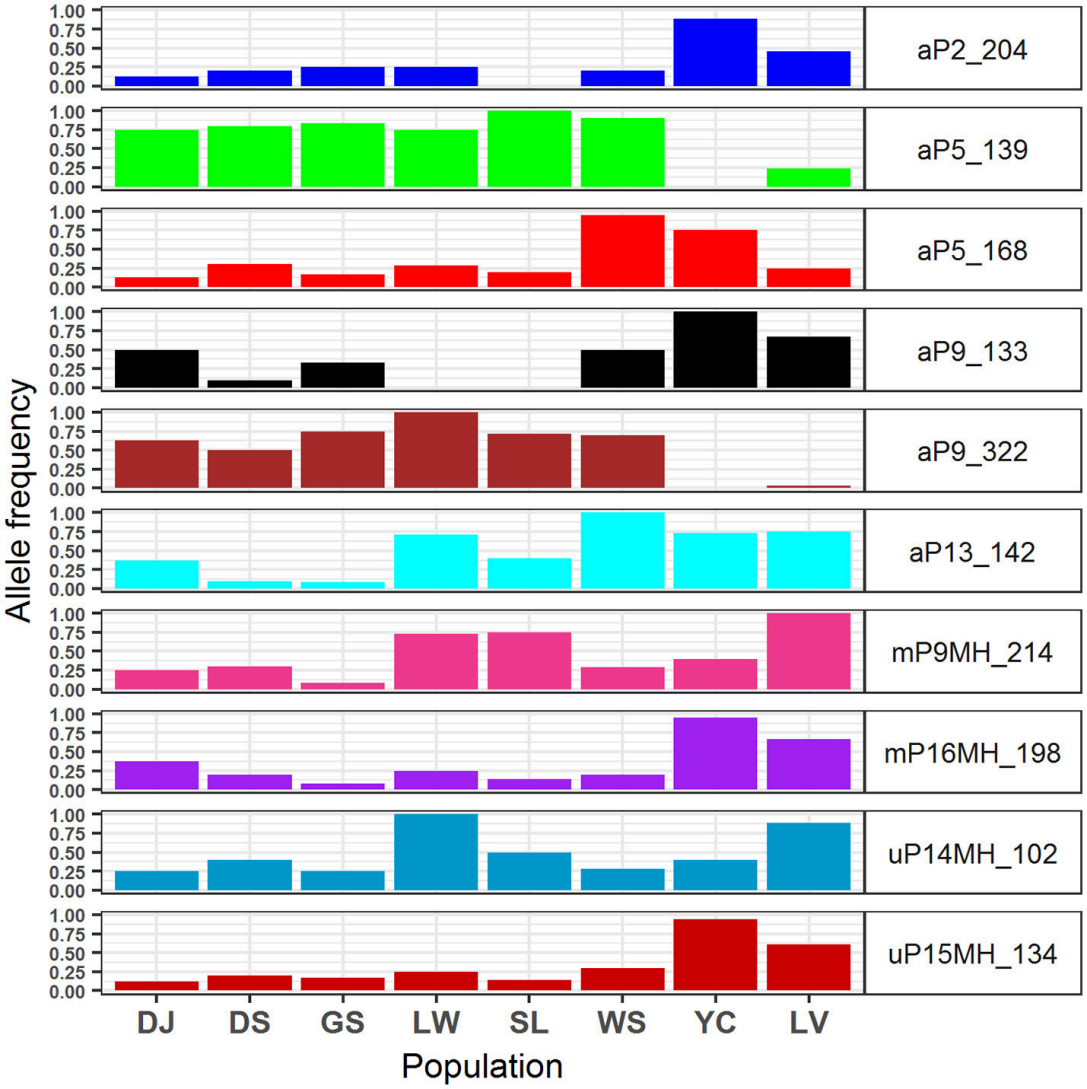
Allele frequencies of the 10 genetic and epigenetic loci strongly associated with environmental variables analyzed using model selection and a model averaging approach of MuMIn.

The 95% CIs for coefficients of environmental covariates which did not overlap with zero suggest individual environmental variables acted on potentially adaptive genetic and epigenetic loci (Table 6). Results showed that six, two, and two outlier AFLP, MSAP-m, and MSAP-u loci, respectively, were found to be strongly associated with environmental variables. The most commonly found important environmental variable explaining genetic and epigenetic variations was monthly temperature variation in agreement with the forward selection results (Supplementary Table 7). In the MuMIn results, monthly temperature variation was the most important explanatory factor for five AFLP (aP2_204, aP5_139, aP5_168, aP9_322, and aP13_142), two MSAP-m (mP9MH_214 and mP16MH_198), and one MSAP-u loci (uP15MH_134) (Table 6, Supplementary Table 8). MSAP-u locus uP14MH_102, with relative importance of 1.00, was found to be strongly correlated with NDVI (Supplementary Table 8). AFLP locus aP9_133 was strongly associated with all six environmental variables except monthly temperature variation, all with relative importance of 1.00. Population allele frequencies of the ten loci (six AFLP, two MSAP-m, and two MSAP-u) strongly associated with environmental variable(s) revealed by MuMIn are depicted in Figure 5.

**Table 6:** Environmental variables strongly associated with genetic and epigenetic loci based on the model averaging (ΔAICc ≤ 3) using R package MuMIn.

	**Estimate for the most important environmental variable(s)**		**Marginal-*R*^2^**
	**Variable (coefficient; 95% CI)**	***P***	
**AFLP**			
aP2_204	BIO4 (0.0014; 0.0002, 0.0025)	0.021	0.2878
aP5_139	BIO4 (−00025; −0.0044, −0.0006)	0.009	0.5689
aP5_168	BIO4 (0.0021; 0.0006, 0.0036)	0.007	0.4654
aP9_133	BIO15 (3.8000; 1.8725, 5.7372)	0.0001	0.9950
	NDVI (260.0; 148.5067, 371.4670)	<0.0001	
	PET (−0.0544; −0.0735, −0.03521)	<0.0001	
	Aspect (−0.0538; −0.0778, −0.2973)	<0.0001	
	Slope (6.458; 3.6299, 9.2863)	<0.0001	
aP9_322	BIO4 (−0.0029; −0.0055, −0.0003)	0.0287	0.2010
aP13_142	BIO4 (0.0022; 0.0007, 0.0037)	0.0037	0.4357
**MSAP-m**			
mP9MH_214	BIO4 (0.0019; 0.0002, 0.0036)	0.026	0.4257
mP16MH_198	BIO4 (0.0023; 0.0011, 00034)	<0.0001	0.4467
**MSAP-u**			
uP14MH_102	NDVI (−27.73; −48.4894, −6.9726)	0.0089	0.7567
uP15MH_134	BIO4 (0.0017; 0.0006, 0.0028)	0.0032	0.3787

*The models listed in each analysis are a 95% confidence set identified on the basis of AICc. The coefficient given for the most important environmental variable(s) is the model averaging coefficient, and the confidence intervals are confidence intervals conditional on the total set of parsimonious (ΔAICc ≤ 3) models that were considered. Marginal-R^2^ given indicates the percent variation of either genetic or epigenetic loci accounted for by the most important environmental variable(s)*.

Within each lineage, the 32 potential selective outliers were subjected to two-locus exact test for LD (Supplementary Table 9). Strong LD was found between AFLP loci and also between AFLP and MSAP-m, between AFLP and MSAP-u, and between MSAP-m and MSAP-u. Interestingly, strong LD between AFLP and MSAP loci were essentially found within Taiwanese and Vietnamese lineages for loci that were revealed to be strongly associated with environmental variable(s) using MuMIn (Table 6, Supplementary Table 9).

## Discussion

### Interplay between DNA sequence divergence and methylation in taiwania

DNA sequence divergence, random neutral events, or environmental gradients may play roles, alone or together, in contributing to epigenetic variation (Richards, 2006). In Taiwania, we found significant Mantel correlations between genetic and epigenetic variations and between methylated and unmethylated types of epigenetic variation. These results indicate not only partial dependence of epigenetic variation on DNA sequence variation, but also interactions between the two types of epigenetic variation, suggesting that such variation may mainly belong to the facilitated type of epigenetic variation proposed by Richards (2006). Interplay between sequence changes and methylation modifications could be involved in Taiwania lineage adaptive divergence as further suggested by strong LD (Supplementary Table 9) between AFLP and MSAP loci that have potentially evolved under selection (Tables 5, 6). Our results suggest environmental gradients such as monthly temperature variation (Table 6) could have critical impact on DNA sequence changes and the downstream influence on methylation pattern of potentially the same genes or linked loci, particularly within Taiwanese and Vietnamese lineages (Supplementary Table 9), which is consistent with other studies that suggest genetic-epigenetic interconnections (Richards, 2006, 2008; Bossdorf et al., 2008; Verhoeven et al., 2010; Schmitz et al., 2013; Silveira et al., 2013).

However, independence between epigenotypes and genotypes cannot be excluded, and a part of the genetic and epigenetic variation could be derived from drift-driven stochastic errors generated, respectively, via random sequence mutation and during the mitotic process independent of sequence divergence (Richards, 2008). In addition, epigenetic variation can be generated in parallel with no functional link to genetic variation (Richards et al., 2010).

### Higher diversity and divergence suggest taiwanese taiwania could be the oldest extant taiwania lineage

The Taiwanese and the Yunnan-Myanmar lineage diverged 3.23–3.41 Mya, whereas the Yunnan-Myanmar and the Vietnamese lineage diverged 1.0–1.39 Mya (Chou et al., 2011). Gene flow between conspecific island-mainland lineages is possible (e.g., between *Cu. lanceolata* and *Cu. konishii*, Hwang et al., 2003; Chung et al., 2004) and may result in lower than expected level of allopatric isolation (Otte and Endler, 1989; Losos and Ricklefs, 2009). In the present study, several lines of evidence (LEA, DAPC, and STRUCTURE; Figures 3, 4, Supplementary Figure 4), in agreement with the results based on cpDNA sequence variation (Chou et al., 2011), displayed distinct differentiation of Taiwanese individuals from those of mainland Asia lineages. Apparent incomplete lineage sorting of ancestral polymorphism between Yunnan-Myanmar and Vietnamese lineages was found based on the LEA, DAPC, and STRUCTURE results (Figures 3, 4, Supplementary Figure 4), however, a much lesser extent of incomplete lineage sorting of ancestral polymorphism between island and mainland Asia lineages was observed. Incomplete lineage sorting of ancestral polymorphism between the two mainland Asia lineages is also supported by the lower, albeit significant levels of genetic and epigenetic differentiation (Supplementary Table 6), in contrast to higher *F*_ST_ levels between island and mainland Asia lineages (Supplementary Table 6). Our results ruled out the hypotheses of recent divergence and extensive gene flow between island and mainland Asia lineages and supports an older divergence between the Taiwanese and the other two lineages as revealed in cpDNA study (Chou et al., 2011).

Our results suggest that most of the genetic and epigenetic variations in Taiwanese lineage is likely to have accumulated since divergence from the mainland Asia lineages 3.23–3.41 Mya. Taiwania in Taiwan could be the oldest extant lineage due to the higher values of the indices, including the percentage of polymorphism, average number of AFLP private allele, and average genetic and epigenetic diversity found in this lineage when compared to the mainland Asia lineages (Tables 1, 2). Although samples from south of Gaoligonshan were not surveyed for genetic and epigenetic variations in the present study, a much lower level of genetic diversity, based on inter-simple sequence repeats, was found compared with the Gaoligoshan population (*H*_E_, 0.0052 vs. 0.0316; Li Z.-C. et al., 2008). It is likely that Gaoligonshan population may harbor the main reservoir of genetic variation along the Yunnan-Myanmar border. Moreover, existing low levels of genetic diversity in mainland Asia lineages could be a consequence of small population sizes due to historical or recent bottlenecks and/or human disturbances. Nonetheless, the examined Gaoligonshan population may not be representative for Taiwania inhabiting large geographic area along the Yunnan-Myanmar border, therefore, evidence provided in the present study may not be conclusive and more complete sampling in the area is necessary for future research.

### The strong population nonadaptive and environmentally dependent between-lineage-divergence in taiwania

The question of what determines inter- and intra-specific divergence has long been a central issue in evolutionary biology. It is not uncommon that populations of conifers evolved by responding to environments (e.g., Mimura and Aitken, 2010; Grivet et al., 2011; Chen et al., 2012; Fang et al., 2013; Shih et al., 2018). However, neutral evolution could also have a strong effect on population and species divergence (Lynch, 2007; Wang et al., 2017). Two hypotheses might explain how populations diversify: selection driven and drift associated divergence (Leffler et al., 2012). Drift induced divergence is commonly associated with past demographic events (Barton, 1996; Comes et al., 2008) and selection driven divergence is emphasized by the strong association of genetic variation with environmental gradients (Schluter, 2001, 2009; Via, 2009; Barton, 2010). Our results of AMOVA, *F*_ST_, and θ^II^ displayed overall significant differentiation (Table 3) among Taiwania populations, however, most pairwise *F*_ST_ between Taiwanese populations showed no significant AFLP differentiation (Supplementary Table 6). In addition, no potential selective outliers were detected using genome scan methods and also no correlation of allele frequencies of genetic and epigenetic loci with environmental gradients was found using Samβada. These results suggest that strong neutral drift acted on population differentiation within Taiwan.

In the present study, environmentally dependent selection between Taiwania lineages could be weak because only a small fraction of variation was explained purely by environments (Table 4), and also only a minor portion of AFLP and MSAP loci were identified as potential selective outliers (Table 5). In addition, only ten of the 32 potential selective outliers detected (six AFLP and two each for MSAP-m and MSAP-u) were found to be associated strongly with specific environmental factors (Table 6). These results also suggest that strong nonadaptive forces played important roles in shaping gene pool structure between lineages.

Substantially large amounts of unaccounted genetic and epigenetic variation (fraction [d], Table 4), which is typical for study integrating environmental features in multivariate analysis (Cottenie, 2005), suggest that fine-scale landscape heterogeneity among habitats might have played a crucial role in shaping divergence between lineages. The fine-scale landscape in habitats of the three lineages can differ greatly, albeit only two land cover types were revealed based on estimation using MODIS product MCD12Q1 of 2013 (Figure 1). Along the Yunnan-Myanmar border, Taiwania is associated mainly with *Tsuga dumosa, Alnus nepalensis, Pinus yunnanensis, Manglietia insignis*, and *Cyclobalanopsis gambleana* (He et al., 2015). In northern Vietnam it is mainly associated with *Fokienia hodginsii* with the forest being otherwise comprised of Fagaceae, Lauraceae, and Magnolia (Farjon and Thomas, 2007). In Taiwan it is associated with other conifers including *Chamaecyparis obtusa* var. *formosana, Ch. formosensis*, and *Cu. konishii*, but may also occur as scattered trees surrounded by broadleaved species of Fagaceae and Lauraceae (Fang et al., 1990; Huang et al., 1994). The differences in floristic compositions between habitats of Taiwania lineages suggest that landscape heterogeneity might have contributed to the large unaccounted genetic and epigenetic variations found using variation partitioning (Table 4). In addition, genetic and epigenetic variations of Taiwania may also be influenced by historical biogeographical processes (Ricklefs and Jenkins, 2011; Chen et al., 2017; Herrera et al., 2017; Richards et al., 2017).

In the present study, a large portion of total explained variation (fraction [a + b + c]) was attributed to geographically-structured environmental variables (Table 4), suggesting that interaction of environment and geography had crucial influence on genetic and epigenetic variations between lineages. The habitats of the lineages differ greatly in the complexity of floristic compositions, and local environments cannot be fully represented by the six retained environmental variables (Supplementary Table 2). Habitat association of genetic and epigenetic diversity with local environments is not uncommon (e.g., Lira-Medeiros et al., 2010; Latzel et al., 2013; Ma et al., 2018; Zoldoš et al., 2018) and it is likely that unmeasured environmental variables, constituting fine-scale microclimates, could also have impact on genetic and epigenetic variations, and may have played important roles in shaping Taiwania lineage divergence.

### Monthly temperature variation and NDVI could be the most important environmental variables driving taiwania lineage divergence

Using the 32 potential outliers (Table 5), results of forward selection showed environmental variation, including monthly temperature variation, NDVI, and aspect, have an impact on outlier genetic and epigenetic variations (Supplementary Table 7). Moreover, analysis with model selection and a model averaging approach using MuMIn revealed strong correlations of nine potential selective outliers with either monthly temperature variation or NDVI (Table 6, Supplementary Table 8).

The values of monthly temperature variation were larger in Yunnan-Myanmar and Vietnamese lineage compared with populations of Taiwanese lineage (Supplementary Table 2). Allele frequencies of two outlier AFLP loci (aP5_139 and aP9_322) associated strongly with monthly temperature variation (Table 6), were found to be lower in the Vietnamese lineage and absent in the Yunnan-Myanmar lineage compared with Taiwanese populations (Figure 5). The accumulation of allele frequencies of aP5_139 and aP9_322 may have been driven by smaller fluctuations in temperature range in Taiwanese populations. In contrast, comparatively higher values of allele frequency of two AFLP (aP2_204 and aP9_133), one MSAP-m (mP16MH_198), and one MSAP-u (uP15MH_134) loci were found in Yunnan-Myanmar and Vietnamese lineages compared with Taiwanese populations. These loci were also found to be associated strongly with monthly temperature variation. Hence, differential values of allele frequency in genetic and epigenetic loci associated with monthly temperature variation suggest that different genetic and epigenetic loci responded differently to different amplitudes of temperature fluctuation in Taiwania, and they could have evolved by either positive or negative directional selection.

Moreover, genetic variation might have influenced the downstream methylation changes in response to monthly temperature variation as suggested by strong LD between pairs of genetic and epigenetic loci within Taiwanese lineage (aP5_139–mP9MH_214 and aP9_322–mP9MH_214) and within Vietnamese lineage (aP2_204–uP15MH_134, aP9_133–mP9MH_214, and aP9_322–uP15MH_134) (Supplementary Table 9) (Richards et al., 2017). However, in most Taiwanese populations, allele frequencies of the epigenetic loci that were identified as potentially having evolved under selection was found to be low (Figure 5). The homogenizing effect due to frequent gene flow as revealed by low and non-significant pairwise *F*_ST_, particularly based on AFLP (Supplementary Table 6), could have a damping impact particularly on epigenetic loci despite overall environmental heterogeneity among Taiwanese populations. In addition, inbreeding within all Taiwania populations was likely (Supplementary Table 4) and significant non-random associations between loci within each population indicated by *I*_A_ and *r*D (Table 2). These results suggest that mating between closely related individuals, probably due to population bottlenecks, may result in loss of alleles due to random drift (Ellstrand and Elam, 1993).

NDVI is a measure of surface coverage activity and has been shown to be correlated with intraspecific (Huang et al., 2015a; Chen et al., 2017) and interspecific adaptive divergence (Nakazato et al., 2010; Huang et al., 2015a). NDVI represents the level of vegetation greenness, a proxy to photosynthetic activity, and may be an influential factor acting on epigenetic variation (uP14MH_102) in response to interactions with other species in a local ecological community (Violle et al., 2012). Moreover, lower values of NDVI may correspond to higher allele frequency of uP14MH_102 in the population Liwusi of Taiwanese Taiwania and the Vietnamese population (Supplementary Table 2 and Figure 5). However, the relationship can be complex, intertwining with other habitat characteristics because low NDVI value was also found for population in Yunnan-Myanmar but with low uP14MH_102 allele frequency. The amount of evergreen conifer foliage may change with season (Gamon et al., 2016) or with disturbance. In Gaoligongshan (Yunnan-Myanmar) Taiwania thrives in unstable habitats subject to frequent landslides, and is dependent on disturbances or gap regeneration for recruitment (He et al., 2015). Under these conditions the extent of the vegetation cover may be dramatically altered. In contrast, habitats of Taiwanese populations have relatively stable conditions in mixed forests with open canopy (Fang et al., 1990; Huang et al., 1994). Such stable conditions are also thought to have been typical for the Vietnamese populations where it grows in dense and tall mixed forest stands and is associated mainly with *F. hodginsii* (Sano et al., 2009).

Our results suggest not only the individual environmental effects on genetic and epigenetic variations in Taiwania, but also that a probable combinatorial effect of certain environmental variables on genetic variation of an AFLP locus (aP9_133 locus). Higher allele frequency of aP9_133 was found in Yunnan-Myanmar and Vietnamese lineages in contrast to Taiwanese lineage (Figure 5), and aP9_133 was found to be strongly associated with a combinatorial effect of all six environmental variables excluding monthly temperature variation (Table 6). This AFLP locus was found to be linked strongly with MSAP mP9MH_214 (within Taiwanese lineage) and uP14MH_102 (within Vietnamese lineage) (Supplementary Table 9). No strong LD with epigenetic variation was found within Yunnan-Myanmar lineage, albeit high aP9_133 allele frequency was also found. This could be due to the smaller sample size collected (Excoffier and Slatkin, 1995). These results suggest that individual environmental variables may not act independently in shaping geographic distributions of genetic and epigenetic variations, and interdependencies of different environmental variables may exert direct and indirect effects on genetic and also the downstream epigenetic changes in Taiwania (Richards, 2006, 2008; Bossdorf et al., 2008; Verhoeven et al., 2010; Schmitz et al., 2013; Silveira et al., 2013).

## Conclusions

Conservation genetics emphasizes the maintenance of the evolutionary capability of species adaptations to varying environments (Frankham et al., 2012). Genetic adaptation to environmental changes is crucial for the conservation and survival of species. In addition, ecologists are increasingly interested in epigenetic adaptations to different environments. Both genetic and epigenetic variations may be sources of variation which play a role in adapting to environmental heterogeneity and hence are important for understanding how environments shape natural population diversity in the face of global environmental changes. In the present study, we found that neutral evolution could have played a predominant role in shaping Taiwania population and lineage divergence. However, either genetic or epigenetic, or a combination may be driving lineage divergence in response to ecological niche differentiation (Li Y. et al., 2008; Herrera and Bazaga, 2010; Lira-Medeiros et al., 2010; Richards et al., 2012; Latzel et al., 2013; Huang et al., 2015a,b; Herrera et al., 2017; Ma et al., 2018) which may trigger speciation (Flatscher et al., 2012; Fernández-Mazuecos and Glover, 2017). Nonetheless, the drift-driven genetic and epigenetic variations may also have potential in adaptation to future climate changes in the different geographical regions that host the main extant Taiwania lineages.

## Author contributions

S-YH proposed, funded, and designed the research. PT, J-DC, and C-NW collected samples. Y-SL performed research. S-YH, Y-SL, and C-TC analyzed data. S-YH, Y-SL, C-TC, PT, and C-NW wrote the paper. All authors have read and approved the final manuscript.

### Conflict of interest statement

The authors declare that the research was conducted in the absence of any commercial or financial relationships that could be construed as a potential conflict of interest.

## Supplementary material

Supplementary Figure 1 Correlation coefficients between environmental variables. **(A)** The eight retained environmental variables when only Taiwanese populations were compared and **(B)** the six retained environmental variables when Taiwania lineages were compared.

Supplementary Figure 2 Variance inflation factor (VIF) of environmental variables. Environmental variables were classified into bioclimate, ecology, and topology categories, and VIF calculated separately. VIFs were reported when only Taiwanese populations were considered (blue bars) or all populations were considered (pink bars).

Supplementary Figure 3 Indices for evaluation of clustering scenarios. **(A)** Mean log probability (LnP(D)), **(B)** change in the log probability (ΔK), **(C)** symmetric similarity coefficient (SSC) obtained from analysis using STRUCTURE, and **(D)** minimal cross-entropy (CE) analyzed using LEA.

Supplementary Figure 4 Individual assignments analyzed using STRUCTURE for clustering scenarios of *K* = 2–4. The subpanels display results analyzed based on the **(A)** AFLP, **(B)** MSAP-m, and **(C)** MSAP-u datasets, respectively. See Table 1 for abbreviations of the eight populations of Taiwania.

Supplementary Table 1 Primer combinations, number of markers, and error rate per locus in AFLP and MSAP techniques.

Supplementary Table 2 Site environmental variables of the eight populations of Taiwania. See Table 1 for abbreviations of the eight populations of Taiwania.

Supplementary Table 3 Pairwise comparisons of environmental differences for Taiwania populations occurring in Taiwan based on the eight retained environmental variables using permutational multivariate analysis of variance.

Supplementary Table 4 Results of *post-hoc* test for multiple comparisons of mean unbiased expected heterozygosity between lineages and between populations of Taiwania using linear mixed effect model. Lineage and population were treated as fixed effects and locus as a random effect.

Supplementary Table 5 The level of differentiation and inbreeding estimated using HICKORY based on genetic and epigenetic markers of Taiwania.

Supplementary Table 6 Pairwise FST between lineages and between populations of Taiwania using ARLEQUIN with 10,000 permutations.

Supplementary Table 7 Results of forward selection based on the 15 AFLP, 4 MSAP-m, and 13 MSAP-u outliers potentially evolved under selection. Environmental variables were classified into three categories, i.e., bioclimate, ecology, and topology, and analyzed separately.

Supplementary Table 8 The relative importance of the environmental variables acting as selective drivers of potentially genetic and epigenetic variations evaluating using AICc values (ΔAICc ≤ 3) using R package MuMIn. The models listed in each analysis are a 95% confidence set identified on the basis of AICc. Relative importance denotes the probability that a given environmental variable, among all parsimonious models considered, is in the model best approximating to the best model. All bold predictor variables have confidence intervals that do not include zero.

Supplementary Table 9 Linkage disequilibrium between outlier genetic and epigenetic loci using a two locus exact test implemented in ARLEQUIN, and significance determined by 10,000 permutations.

Data Sheet 1 Comma-separated value file for scored 1413 AFLP markers of the eight populations (*n* = 102) of Taiwania. The first three columns recorded the information of sample ID, lineage, and population. The rest of columns represent presence/absence of markers.

Data Sheet 2 Comma-separated value file for scored 456 MSAP-m markers of the eight populations (*n* = 102) of Taiwania. The first three columns recorded the information of sample ID, lineage, and population. The rest of columns represent presence/absence of markers.

Data Sheet 3 Comma-separated value file for scored 289 MSAP-u markers of the eight populations (*n* = 102) of Taiwania. The first three columns recorded the information of sample ID, lineage, and population. The rest of columns represent presence/absence of markers.
