# Supplementary figures and images for: The Contribution of Neutral and Environmentally Dependent Processes in Driving Population and Lineage Divergence in Taiwania (Taiwania cryptomerioides)
Source: Front Plant Sci. 2018 Aug 8;9:1148. doi: 10.3389/fpls.2018.01148 (PMC6092574; doi:10.3389/fpls.2018.01148)

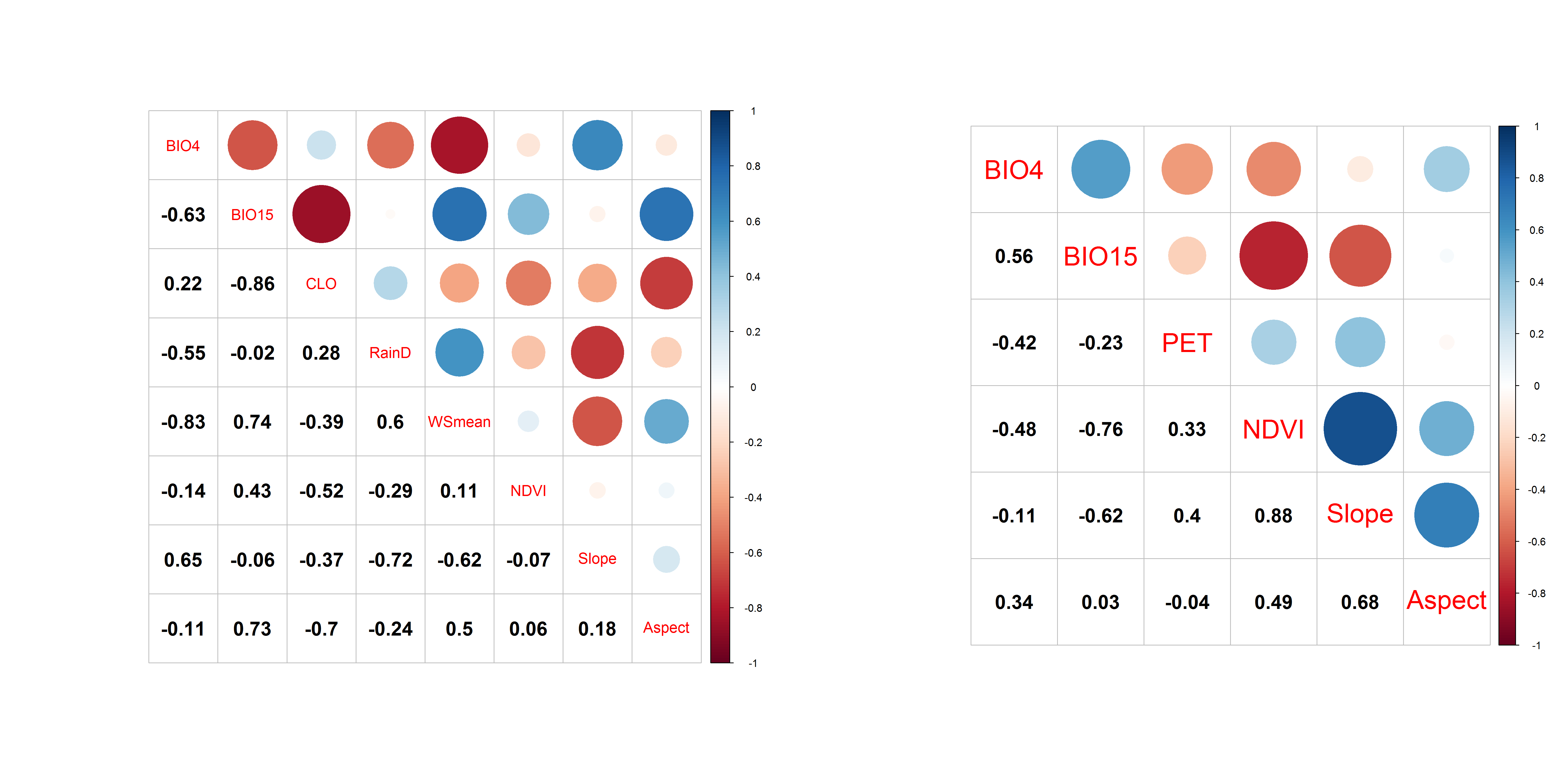

Supplement: Supplementary Figure 1 — Correlation coefficients between environmental variables. (A) The eight retained environmental variables when only Taiwanese populations were compared and (B) the six retained environmental variables when Taiwania lineages were compared. [file Image_1.TIF]

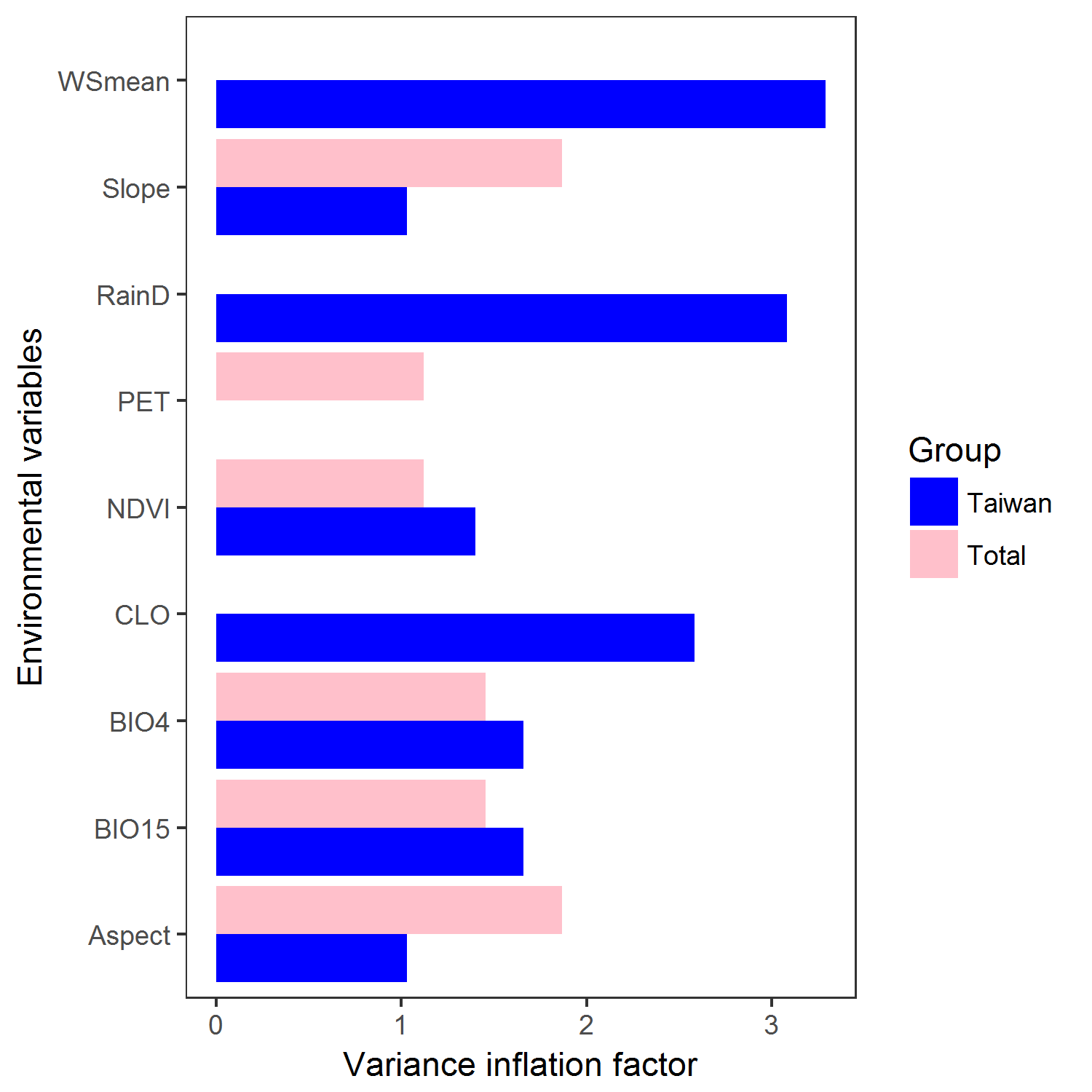

Supplement: Supplementary Figure 2 — Variance inflation factor (VIF) of environmental variables. Environmental variables were classified into bioclimate, ecology, and topology categories, and VIF calculated separately. VIFs were reported when only Taiwanese populations were considered (blue bars) or all populations were considered (pink bars). [file Image_2.TIF]

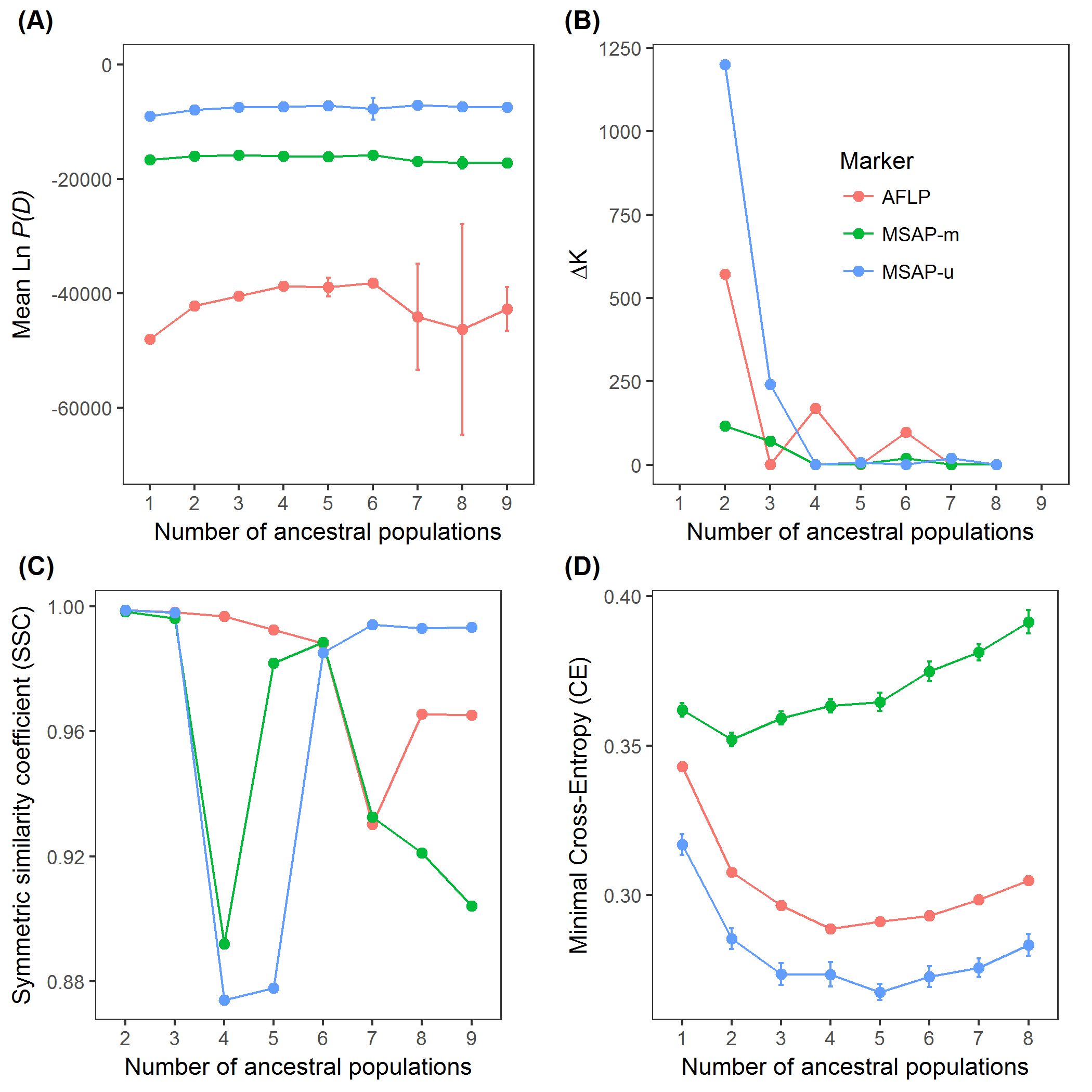

Supplement: Supplementary Figure 3 — Indices for evaluation of clustering scenarios. (A) Mean log probability (LnP(D)), (B) change in the log probability (ΔK), (C) symmetric similarity coefficient (SSC) obtained from analysis using STRUCTURE, and (D) minimal cross-entropy (CE) analyzed using LEA. [file Image_3.TIF]

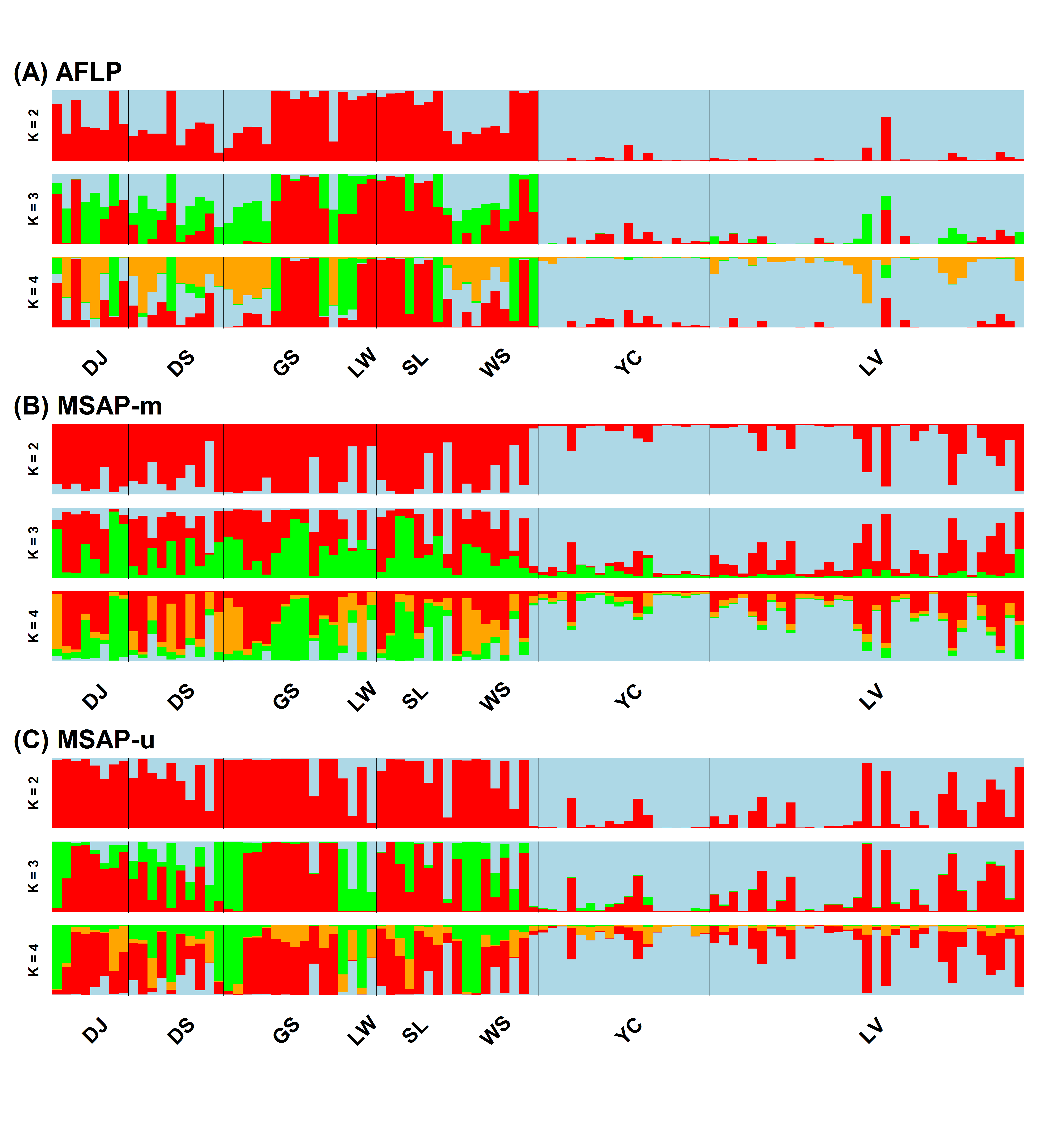

Supplement: Supplementary Figure 4 — Individual assignments analyzed using STRUCTURE for clustering scenarios of K = 2–4. The subpanels display results analyzed based on the (A) AFLP, (B) MSAP-m, and (C) MSAP-u datasets, respectively. See Table 1 for abbreviations of the eight populations of Taiwania. [file Image_4.TIF]
