# Supplementary material for: The Contribution of Neutral and Environmentally Dependent Processes in Driving Population and Lineage Divergence in Taiwania (Taiwania cryptomerioides)
Source: Front Plant Sci. 2018 Aug 8;9:1148. doi: 10.3389/fpls.2018.01148 (PMC6092574; doi:10.3389/fpls.2018.01148)
Supplement: Supplementary Table 1 — Primer combinations, number of markers, and error rate per locus in AFLP and MSAP techniques. [file Table_1.DOCX]

**Supplementary Table 1.** Primer combinations, number of markers, and error rate per locus in AFLP and MSAP techniques.

| **AFLP** | | |  | **MSAP** | | | | |
| --- | --- | --- | --- | --- | --- | --- | --- | --- |
| Primer number and combination | Number of markers | Error rate (%) |  | Primer number and combination | Number of  markers | Error rate (%) | | Combined error rate  (%) |
|  |  |  |  |  |  | *Hpa*II | *Msp*I |  |
| 1 E00ACAGT+M00GTA | 127 | 3.67 |  | 1 E00CTTGC+HM00AG | 51 | 2.98 | 3.65 | 6.42 |
| 2 E00ACAGT+M00GAT | 123 | 3.19 |  | 2 E00CTTGC+HM00AT | 51 | 2.60 | 2.22 | 4.71 |
| 3 E00ACAGT+M00GCA | 142 | 2.69 |  | 3 E00CTTGC+ HM00GT | 47 | 3.06 | 3.33 | 6.19 |
| 4 E00ACGAA+M00GAT | 136 | 13.32 |  | 4 E00CTATA+HM00AG | 70 | 1.85 | 3.10 | 4.84 |
| 5 E00ACAGT+M00GGC | 123 | 3.28 |  | 5 E00CTGAA+HM00AG | 50 | 2.02 | 1.76 | 3.70 |
| 6 E00ACAGT+M00GCG | 148 | 3.86 |  | 6 E00CTGAA+HM00TA | 70 | 1.32 | 2.18 | 3.45 |
| 7 E00ACGAA+M00GCG | 133 | 10.59 |  | 7 E00CTGTC+HM00AG | 55 | 2.41 | 2.39 | 4.69 |
| 8 E00ACGAA+M00GCA | 122 | 9.80 |  | 8 E00CTTGG+HM00AG | 68 | 2.69 | 2.25 | 4.82 |
| 9 E00ACGAA+M00GTA | 128 | 9.34 |  |  |  |  |  |  |
| 10 E00ACATC+M00GTA | 118 | 10.22 |  |  |  |  |  |  |
| 11 E00ACATC+M00GGC | 113 | 10.48 |  |  |  |  |  |  |
| Average | 128.45 | 7.31 |  |  | 57.75 | 2.37 | 2.61 | 4.85 |

E00 (5’-GACTGCGTACCAATTC-3’)

M00 (5’-GATGAGTCCTGAGTAA-3’)

HM00 (*Hpa*II-*Msp*I, 5’-ATCATGAGTCCTGCTCGG-3’)
