# Supplementary material for: The Contribution of Neutral and Environmentally Dependent Processes in Driving Population and Lineage Divergence in Taiwania (Taiwania cryptomerioides)
Source: Front Plant Sci. 2018 Aug 8;9:1148. doi: 10.3389/fpls.2018.01148 (PMC6092574; doi:10.3389/fpls.2018.01148)
Supplement: Supplementary Table 2 — Site environmental variables of the eight populations of Taiwania. See Table 1 for abbreviations of the eight populations of Taiwania. [file Table_2.DOCX]

**Supplementary Table 2.** Site environmental variables of the eight populations of Taiwania. See **Table 1** for abbreviations of the eight populations of Taiwania.

| Environmental variables | Taiwan | | | | | |  | Mainland China |  | Vietnam | VIF |
| --- | --- | --- | --- | --- | --- | --- | --- | --- | --- | --- | --- |
|  | DJ | DS | GS | LW | SL | WS |  | YC |  | LV |  |
| Bioclimate |  |  |  |  |  |  |  |  |  |  |  |
| BIO1 | 107 | 97 | 108 | 113 | 114 | 125 |  | 133 |  | 142 |  |
| BIO2 | 73 | 74 | 80 | 72 | 70 | 80 |  | 107 |  | 71 |  |
| BIO3 | 44 | 44 | 52 | 45 | 41 | 50 |  | 45 |  | 39 |  |
| BIO4*,^†^ | 3274 | 3350 | 2706 | 3179 | 3567 | 2956 |  | 4794 |  | 3941 | 23.83 |
| BIO5 | 179 | 168 | 173 | 183 | 188 | 195 |  | 233 |  | 218 |  |
| BIO6 | 16 | 3 | 20 | 23 | 20 | 35 |  | -4 |  | 39 |  |
| BIO7 | 163 | 165 | 153 | 160 | 168 | 160 |  | 237 |  | 179 |  |
| BIO8 | 137 | 127 | 136 | 148 | 154 | 157 |  | 188 |  | 182 |  |
| BIO9 | 76 | 88 | 83 | 83 | 79 | 96 |  | 78 |  | 97 |  |
| BIO10 | 144 | 134 | 136 | 148 | 154 | 157 |  | 188 |  | 186 |  |
| BIO11 | 62 | 51 | 70 | 69 | 65 | 84 |  | 67 |  | 86 |  |
| BIO12 | 3280 | 3171 | 3247 | 3347 | 2761 | 3344 |  | 1333 |  | 2073 |  |
| BIO13 | 416 | 520 | 665 | 439 | 366 | 590 |  | 282 |  | 473 |  |
| BIO14 | 93 | 49 | 83 | 117 | 96 | 57 |  | 11 |  | 6 |  |
| BIO15*,^†^ | 45 | 63 | 64 | 36 | 39 | 72 |  | 83 |  | 91 | 44.71 |
| BIO16 | 1235 | 1412 | 1501 | 1137 | 1019 | 1623 |  | 758 |  | 1193 |  |
| BIO17 | 321 | 199 | 256 | 406 | 330 | 177 |  | 44 |  | 36 |  |
| BIO18 | 1101 | 1086 | 1488 | 1137 | 1013 | 157 |  | 758 |  | 1184 |  |
| BIO19 | 413 | 321 | 382 | 460 | 376 | 231 |  | 56 |  | 70 |  |
|  |  |  |  |  |  |  |  |  |  |  |  |
| Topological |  |  |  |  |  |  |  |  |  |  |  |
| Slope*,^†^ | 39.2 | 40.2 | 28 | 27.9 | 32.6 | 32.3 |  | 35.9 |  | 13.9 | 191.78 |
| Aspect*,^†^ | 74 | 237.5 | 126.1 | 143.3 | 93.4 | 246.3 |  | 317.8 |  | 67.3 | 38.53 |
|  |  |  |  |  |  |  |  |  |  |  |  |
| Ecological |  |  |  |  |  |  |  |  |  |  |  |
| NDVI*,^†^ | 0.827 | 0.843 | 0.854 | 0.787 | 0.859 | 0.844 |  | 0.782 |  | 0.637 | 41.98 |
| EVI | 0.452 | 0.436 | 0.418 | 0.349 | 0.41 | 0.469 |  | 0.421 |  | 0.355 |  |
| LAI | 5.932 | 6.031 | 4.772 | 2.945 | 5.223 | 6.131 |  | 5.064 |  | 1.525 |  |
| fPAR | 0.899 | 0.899 | 0.885 | 0.725 | 0.916 | 0.908 |  | 0.848 |  | 0.497 |  |
| PET* | 1514 | 1400.7 | 1143.4 | 608.2 | 659.6 | 971.3 |  | 859.8 |  | 917.8 | 21.75 |
| Soil.pH | 4.9 | 4.8 | 5.2 | 5.8 | 4.7 | 4.9 |  |  |  |  |  |
| MI | 245.4 | 261.8 | 214.4 | 151.5 | 257.9 | 243.7 |  |  |  |  |  |
| RH | 78.028 | 78.102 | 77.454 | 78.062 | 78.811 | 78.381 |  |  |  |  |  |
| CLO^†^ | 6.506 | 6.174 | 6.451 | 7.171 | 6.682 | 6.049 |  |  |  |  |  |
| SunH | 135.483 | 147.413 | 144.055 | 127.665 | 132.878 | 148.824 |  |  |  |  |  |
| RainD^†^ | 12.231 | 10.792 | 12.405 | 12.988 | 11.868 | 12.765 |  |  |  |  |  |
| WSmean^†^ | 2.52 | 2.687 | 3.179 | 2.949 | 2.508 | 3.428 |  |  |  |  |  |

*See http://www.worldclim.org/bioclim for explanation of BIO1–BIO19. Aspect (0–360°), slope (0–90°).*

| *NDVI, normalized difference vegetation index; EVI, enhanced vegetation index; LAI, leaf area index; fPAR, fraction of absorbed photosynthetically active radiation; PET, annual total potential evapotranspiration; MI, annual moisture index; RH, relative humidity ; CLO, cloud cover; SunH, time of sunshine ; RainD, number of rainfall days per year; WSmean, mean wind speed.* |
| --- |

**, environmental variables retained when the three Taiwania lineages were considered.*

*^†^, environmental variables retained when only Taiwanese populations were considered.*

*VIF, variance inflation factor.*

*Infinitive VIFs were obtained when all environmental variables were used in the calculation. Infinitive VIFs were also obtained when the eight environmental variables retained when only Taiwanese populations were considered.*

*VIFs reported were those of the six retained environmental variables when Taiwania lineages were considered.*
