# Supplementary material for: The Contribution of Neutral and Environmentally Dependent Processes in Driving Population and Lineage Divergence in Taiwania (Taiwania cryptomerioides)
Source: Front Plant Sci. 2018 Aug 8;9:1148. doi: 10.3389/fpls.2018.01148 (PMC6092574; doi:10.3389/fpls.2018.01148)
Supplement: Supplementary Table 3 — Pairwise comparisons of environmental differences for Taiwania populations occurring in Taiwan based on the eight retained environmental variables using permutational multivariate analysis of variance. [file Table_3.DOCX]

**Supplementary Table 3.** Pairwise comparisons of environmental differences for Taiwania populations occurring in Taiwan based on the eight retained environmental variables using permutational multivariate analysis of variance.

| Pair | *P* value |
| --- | --- |
| DJ-DS | 1 |
| DJ-GS | 0.0021 |
| DJ-LW | 0.0094 |
| DJ-SL | 0.0021 |
| DJ-WS | 1 |
| DS-GS | 1 |
| DS-LW | 1 |
| DS-SL | 1 |
| DS-WS | 0.0021 |
| GS-LW | 0.0021 |
| GS-SL | 1 |
| GS-WS | 0.0021 |
| LW-SL | 0.0021 |
| LW-WS | 1 |
| SL-WS | 0.0021 |

*Significance determined by 999 permutations and a false discovery rate of 5%.*
