# Supplementary material for: The Contribution of Neutral and Environmentally Dependent Processes in Driving Population and Lineage Divergence in Taiwania (Taiwania cryptomerioides)
Source: Front Plant Sci. 2018 Aug 8;9:1148. doi: 10.3389/fpls.2018.01148 (PMC6092574; doi:10.3389/fpls.2018.01148)
Supplement: Supplementary Table 4 — Results of post-hoc test for multiple comparisons of mean unbiased expected heterozygosity between lineages and between populations of Taiwania using linear mixed effect model. Lineage and population were treated as fixed effects and locus as a random effect. [file Table_4.DOCX]

**Supplementary Table 4.** Results of post-hoc test for multiple comparisons of mean unbiased expected heterozygosity between lineages and between populations of Taiwania using linear mixed effect model. Lineage and population were treated as fixed effects and locus as a random effect.

|  | AFLP | | |  | MSAP-m | | |  | MSAP-u | | |
| --- | --- | --- | --- | --- | --- | --- | --- | --- | --- | --- | --- |
| Contrast | Difference | *t* | *P* |  | Difference | *t* | *P* |  | Difference | *t* | *P* |
|  | |  |  |  |  |  |  |  |  |  |  |
| Between lineages | |  |  |  |  |  |  |  |  |  |  |
| YC - TW | -0.1373 | -35.20 | < 0.0001 |  | -0.0302 | -4.51 | < 0.0001 |  | -0.0451 | -4.67 | < 0.0001 |
| YC - LV | -0.0330 | -8.45 | < 0.0001 |  | 0.0164 | 2.45 | 0.0386 |  | 0.0228 | 2.36 | 0.0485 |
| TW - LV | 0.1043 | 26.75 | < 0.0001 |  | 0.0466 | 6.95 | < 0.0001 |  | 0.0680 | 7.03 | < 0.0001 |
|  |  |  |  |  |  |  |  |  |  |  |  |
| Between populations | |  |  |  |  |  |  |  |  |  |  |
| DJ - DS | 0.0244 | 4.13 | 0.001 |  | 0.0244 | 4.13 | 0.001 |  | 0.0044 | 0.32 | 1.000 |
| DJ - GS | 0.0099 | 1.67 | 0.709 |  | 0.0099 | 1.67 | 0.709 |  | 0.0149 | 1.09 | 0.960 |
| DJ - LW | 0.0446 | 7.54 | < 0.0001 |  | 0.0446 | 7.54 | < 0.0001 |  | -0.0653 | -4.75 | 0.000 |
| DJ - SL | 0.0780 | 13.19 | < 0.0001 |  | 0.0780 | 13.19 | < 0.0001 |  | 0.0086 | 0.62 | 0.999 |
| DJ - WS | 0.0164 | 2.77 | 0.102 |  | 0.0164 | 2.77 | 0.102 |  | -0.0037 | -0.27 | 1.000 |
| DJ - YC | 0.1548 | 26.18 | < 0.0001 |  | 0.1548 | 26.18 | < 0.0001 |  | 0.0312 | 2.27 | 0.311 |
| DJ - LV | 0.1218 | 20.61 | < 0.0001 |  | 0.1218 | 20.61 | < 0.0001 |  | 0.0540 | 3.93 | 0.002 |
| DS - GS | -0.0146 | -2.46 | 0.212 |  | -0.0146 | -2.46 | 0.212 |  | 0.0105 | 0.76 | 0.995 |
| DS - LW | 0.0201 | 3.41 | 0.015 |  | 0.0201 | 3.41 | 0.015 |  | -0.0698 | -5.07 | < 0.0001 |
| DS - SL | 0.0536 | 9.06 | < 0.0001 |  | 0.0536 | 9.06 | < 0.0001 |  | 0.0041 | 0.30 | 1.000 |
| DS - WS | -0.0080 | -1.35 | 0.878 |  | -0.0080 | -1.35 | 0.878 |  | -0.0082 | -0.59 | 0.999 |
| DS - YC | 0.1304 | 22.06 | < 0.0001 |  | 0.1304 | 22.06 | < 0.0001 |  | 0.0268 | 1.95 | 0.519 |
| DS - LV | 0.0974 | 16.48 | < 0.0001 |  | 0.0974 | 16.48 | < 0.0001 |  | 0.0496 | 3.61 | 0.008 |
| GS - LW | 0.0347 | 5.87 | < 0.0001 |  | 0.0347 | 5.87 | < 0.0001 |  | -0.0803 | -5.84 | < 0.0001 |
| GS - SL | 0.0681 | 11.52 | < 0.0001 |  | 0.0681 | 11.52 | < 0.0001 |  | -0.0064 | -0.46 | 1.000 |
| GS - WS | 0.0065 | 1.11 | 0.955 |  | 0.0065 | 1.11 | 0.955 |  | -0.0186 | -1.36 | 0.877 |
| GS - YC | 0.1450 | 24.52 | < 0.0001 |  | 0.1450 | 24.52 | < 0.0001 |  | 0.0163 | 1.18 | 0.937 |
| GS - LV | 0.1120 | 18.94 | < 0.0001 |  | 0.1120 | 18.94 | < 0.0001 |  | 0.0391 | 2.84 | 0.085 |
| LW - SL | 0.0334 | 5.66 | < 0.0001 |  | 0.0334 | 5.66 | < 0.0001 |  | 0.0739 | 5.37 | < 0.0001 |
| LW - WS | -0.0282 | -4.76 | 0.000 |  | -0.0282 | -4.76 | 0.000 |  | 0.0616 | 4.48 | 0.000 |
| LW - YC | 0.1103 | 18.65 | < 0.0001 |  | 0.1103 | 18.65 | < 0.0001 |  | 0.0965 | 7.02 | < 0.0001 |
| LW - LV | 0.0773 | 13.07 | < 0.0001 |  | 0.0773 | 13.07 | < 0.0001 |  | 0.1194 | 8.68 | < 0.0001 |
| SL - WS | -0.0616 | -10.42 | < 0.0001 |  | -0.0616 | -10.42 | < 0.0001 |  | -0.0123 | -0.89 | 0.987 |
| SL - YC | 0.0768 | 12.99 | < 0.0001 |  | 0.0768 | 12.99 | < 0.0001 |  | 0.0226 | 1.65 | 0.722 |
| SL - LV | 0.0439 | 7.42 | < 0.0001 |  | 0.0439 | 7.42 | < 0.0001 |  | 0.0455 | 3.31 | 0.022 |
| WS - YC | 0.1384 | 23.41 | < 0.0001 |  | 0.1384 | 23.41 | < 0.0001 |  | 0.0349 | 2.54 | 0.180 |
| WS - LV | 0.1054 | 17.83 | < 0.0001 |  | 0.1054 | 17.83 | < 0.0001 |  | 0.0577 | 4.20 | 0.001 |
| YC - LV | -0.0330 | -5.58 | < 0.0001 |  | -0.0330 | -5.58 | < 0.0001 |  | 0.0228 | 1.66 | 0.713 |

*t, Student’s t statistic.*
