# Supplementary material for: The Contribution of Neutral and Environmentally Dependent Processes in Driving Population and Lineage Divergence in Taiwania (Taiwania cryptomerioides)
Source: Front Plant Sci. 2018 Aug 8;9:1148. doi: 10.3389/fpls.2018.01148 (PMC6092574; doi:10.3389/fpls.2018.01148)
Supplement: Supplementary Table 5 — The level of differentiation and inbreeding estimated using HICKORY based on genetic and epigenetic markers of Taiwania. [file Table_5.DOCX]

**Supplementary Table 5.** The level of differentiation and inbreeding estimated using HICKORY based on genetic and epigenetic markers of Taiwania.

|  | Parameter | | | | | |
| --- | --- | --- | --- | --- | --- | --- |
| Model | D̅ | D̂ | pD | DIC | *f* (95% CI) | *θ*^II^ (SD) |
| Between lineages | |  |  |  |  |  |
|  | |  |  |  |  |  |
| AFLP |  |  |  |  |  |  |
| *Full* | 12123.8 | 9808.1 | 2315.65 | 14439.4 | 0.987 (0.951, 0.999) | 0.135 (0.125, 0.146) |
| *f* = 0 | 12135 | 9772.6 | 2362.44 | 14497.5 |  | 0..085 (0.078, 0.092) |
| *θ* = 0 | 22080.1 | 20891.2 | 1188.88 | 23269 | 0.998 (0.994, 0.999) |  |
| *f* free | 12816.5 | 9782.78 | 3033.68 | 15850.1 | 0.498 (0.002, 0.973) | 0.148 (0.123, 0.177) |
|  |  |  |  |  |  |  |
| MSAP-m |  |  |  |  |  |  |
| *Full* | 4661.66 | 3916.73 | 744.93 | 5406.59 | 0.905 (0.162, 0.998) | 0.047 (0.030, 0.057) |
| *f* = 0 | 4664.09 | 3910.01 | 754.08 | 5418.16 |  | 0.027 (0.027, 0.032) |
| *θ* = 0 | 6033.73 | 5631.17 | 402.57 | 6436.3 | 0.997 (0.988, 0.999) |  |
| *f* free | 4736.3 | 3796.68 | 939.62 | 5675.92 | 0.503 (0.027, 0.973) | 0.055 (0.041, 0.073) |
|  |  |  |  |  |  |  |
| MSAP-u |  |  |  |  |  |  |
| *Full* | 2477.24 | 1989.36 | 487.88 | 2965.12 | 0.977 (0.910, 0.999) | 0.135 (0.134, 0159) |
| *f* = 0 | 2480.48 | 1982.12 | 498.36 | 2978.85 |  | 0.085 (0.070, 0.103) |
| *θ* = 0 | 4411.37 | 4172.54 | 238.83 | 5650.2 | 0.994 (0.978, 0.999) |  |
| *f* free | 2580.93 | 2989.04 | 591.90 | 3172.83 | 0.510 (0.022, 0.975) | 0.141 (0.112, 0.175) |
|  |  |  |  |  |  |  |
| *Between populations* | |  |  |  |  |  |
|  | |  |  |  |  |  |
| AFLP |  |  |  |  |  |  |
| *Full* | 24617.8 | 20241.9 | 4375.85 | 28993.6 | 0.987 (0.950,0.997) | 0.125 (0.119, 0.132) |
| *f* = 0 | 24639 | 20121 | 4517.81 | 29156.8 |  | 0.080 (0.076, 0.085) |
| *θ* = 0 | 37191.8 | 36003.3 | 1188.45 | 38380.2 | 0.998 (0.994, 0.999) |  |
| *f* free | 24931.1 | 19867.5 | 5063.53 | 29994.6 | 0.498 (0.027, 0.974) | 0.125 (0.110, 0.140) |
|  |  |  |  |  |  |  |
| MSAP-m |  |  |  |  |  |  |
| *Full* | 8760.43 | 7567.8 | 1192.63 | 9953.07 | 0.787 (0.058, 0.996) | 0.053 (0.033, 0.064) |
| *f* = 0 | 8758.02 | 7538.87 | 1219.15 | 9977.16 |  | 0.033 (0.028, 0.037) |
| *θ* = 0 | 10832.8 | 10430.7 | 402.131 | 11234.9 | 0.997 (0.987, 0.999) |  |
| *f* free | 8766.14 | 7314.91 | 1451.23 | 10217.4 | 0.493 (0.022, 0.975) | 0.056 (0.046, 0066) |
|  |  |  |  |  |  |  |
| MSAP-u |  |  |  |  |  |  |
| *Full* | 4567.68 | 3714.93 | 852.748 | 5420.43 | 0.983 (0.935, 0.999) | 0.148 (0.131, 0.165) |
| *f* = 0 | 4579.74 | 3707.58 | 872.168 | 5451.91 |  | 0.009 (0.081, 0.104) |
| *θ* = 0 | 7373.3 | 7134.58 | 238.713 | 7612.01 | 0.994 (0.977, 0.999) |  |
| *f* free | 4634.35 | 3684.42 | 949.936 | 5584.29 | 0.496 (0.027, 0.974) | 0.137 (0.116, 0.158) |

*θ^II^, is the best Bayesian inference estimate of the proportion of genetic diversity due to differences among populations, and is an analogue to F_ST_.*

*D̅, is a measure of how well the model fits the data (smaller values indicate a better fit).*

*D̂, is a measure of how well the best point estimate fits the data.*

*pD, is a measure of model complexity, i.e., the effective number of parameters being estimated (pD = D̅-D̂).*

*DIC, deviance information criterion.*

*f, an estimate of F_IS_, inbreeding within a population*
