# Supplementary material for: The Contribution of Neutral and Environmentally Dependent Processes in Driving Population and Lineage Divergence in Taiwania (Taiwania cryptomerioides)
Source: Front Plant Sci. 2018 Aug 8;9:1148. doi: 10.3389/fpls.2018.01148 (PMC6092574; doi:10.3389/fpls.2018.01148)
Supplement: Supplementary Table 6 — Pairwise FST between lineages and between populations of Taiwania using ARLEQUIN with 10,000 permutations. [file Table_6.DOCX]

**Supplementary Table 6.** Pairwise *F*_ST_ between lineages and between populations of Taiwania using ARLEQUIN with 10,000 permutations.

|  | Pairwise *F*_ST_ (bottom diagonal) and *P* (upper diagonal) values | | | | | | | |
| --- | --- | --- | --- | --- | --- | --- | --- | --- |
| Between lineages | |  |  |  |  |  |  |  |
| AFLP | Taiwan | Yunnan-Myanmar | Vietnam |  |  |  |  |  |
| Taiwan |  | < 0.0001 | < 0.0001 |  |  |  |  |  |
| Yunnan-Myanmar | 0.16789 |  | < 0.0001 |  |  |  |  |  |
| Vietnam | 0.14933 | 0.08067 |  |  |  |  |  |  |
|  |  |  |  |  |  |  |  |  |
| MSAP-m | Taiwan | Yunnan-Myanmar | Vietnam |  |  |  |  |  |
| Taiwan |  | < 0.0001 |  |  |  |  |  |  |
| Yunnan-Myanmar | 0.08231 |  | < 0.0001 |  |  |  |  |  |
| Vietnam | 0.04589 | 0.04421 |  |  |  |  |  |  |
|  |  |  |  |  |  |  |  |  |
| MSAP-u | Taiwan | Yunnan-Myanmar | Vietnam |  |  |  |  |  |
| Taiwan |  | < 0.0001 | < 0.0001 |  |  |  |  |  |
| Yunnan-Myanmar | 0.21324 |  | < 0.0001 |  |  |  |  |  |
| Vietnam | 0.14043 | 0.10291 |  |  |  |  |  |  |
|  |  |  |  |  |  |  |  |  |
| Between populations | |  |  |  |  |  |  |  |
|  |  |  |  |  |  |  |  |  |
| AFLP | DJ | DS | GS | LW | SL | WS | YC | LV |
| DJ |  | 0.3237 | 0.7077 | 0.1397 | 0.0365 | 0.8001 | < 0.0001 | < 0.0001 |
| DS | 0.00492 |  | 0.1329 | 0.0084 | 0.0007 | 0.2252 | < 0.0001 | < 0.0001 |
| GS | 0 | 0.01842 |  | 0.1855 | 0.0634 | 0.4558 | < 00001 | < 0.0001 |
| LW | 0.03319 | 0.11144 | 0.02952 |  | 0.1959 | 0.1088 | < 0.0001 | < 0.0001 |
| SL | 0.05882 | 0.15126 | 0.05095 | 0.02495 |  | 0.0132 | < 0.0001 | < 0.0001 |
| WS | 0 | 0.00871 | 0 | 0.04513 | 0.07598 |  | < 0.0001 | < 0.0001 |
| YC | 0.22538 | 0.20415 | 0.2233 | 0.41819 | 0.39774 | 0.21772 |  | < 0.0001 |
| LV | 0.17777 | 0.13878 | 0.18644 | 0.35509 | 0.36068 | 0.17489 | 0.08067 |  |
|  |  |  |  |  |  |  |  |  |
| MSAP-m | DJ | DS | GS | LW | SL | WS | YC | LV |
| DJ |  | 0.0803 | 0.5281 | 0.0113 | 0.9876 | 0.1914 | < 0.0001 | < 0.0001 |
| DS | 0.01458 |  | 0.0099 | 0.0145 | 0.1441 | 0.4131 | < 0.0001 | < 0.0001 |
| GS | 0 | 0.02238 |  | 0.0018 | 0.5695 | 0.0258 | < 0.0001 | < 0.0001 |
| LW | 0.05474 | 0.05656 | 0.06556 |  | 0.0068 | 0.0072 | 0.0003 | < 0.0001 |
| SL | 0 | 0.01161 | 0 | 0.06991 |  | 0.0117 | < 0.0001 | < 0.0001 |
| WS | 0.00911 | 0.00175 | 0.02002 | 0.05735 | 0.02607 |  |  | < 0.0001 |
| YC | 0.10342 | 0.07676 | 0.12311 | 0.10273 | 0.10995 | 0.08216 |  | < 0.0001 |
| LV | 0.05743 | 0.04584 | 0.08617 | 0.11384 | 0.07576 | 0.05389 | 0.04421 |  |
|  |  |  |  |  |  |  |  |  |
| MSAP-u | DJ | DS | GS | LW | SL | WS | YC | LV |
| DJ |  | 0.0174 | 0.3375 | 0.0021 | 0.2935 | 0.2472 | < 0.0001 | < 0.0001 |
| DS | 0.01458 |  | 0.0006 | 0.0060 | < 0.0001 | 0.3766 | < 0.0001 | < 0.0001 |
| GS | 0 | 0.02238 |  | 0.0007 | 0.5136 | 0.0108 | < 0.0001 | < 0.0001 |
| LW | 0.05474 | 0.05656 | 0.06556 |  | 0.0029 | 0.0036 | < 0.0001 | < 0.0001 |
| SL | 0 | 0.01161 | 0 | 0.06991 |  | 0.0070 | < 0.0001 | < 0.0001 |
| WS | 0.00911 | 0.00175 | 0.02002 | 0.05735 | 0.02607 |  | < 0.0001 | < 0.0001 |
| YC | 0.10342 | 0.07676 | 0.12311 | 0.10273 | 0.10995 | 0.08216 |  | < 0.0001 |
| LV | 0.05743 | 0.04584 | 0.08617 | 0.11384 | 0.07576 | 0.05389 | 0.04421 |  |
