# Supplementary material for: The Contribution of Neutral and Environmentally Dependent Processes in Driving Population and Lineage Divergence in Taiwania (Taiwania cryptomerioides)
Source: Front Plant Sci. 2018 Aug 8;9:1148. doi: 10.3389/fpls.2018.01148 (PMC6092574; doi:10.3389/fpls.2018.01148)
Supplement: Supplementary Table 7 — Results of forward selection based on the 15 AFLP, 4 MSAP-m, and 13 MSAP-u outliers potentially evolved under selection. Environmental variables were classified into three categories, i.e., bioclimate, ecology, and topology, and analyzed separately. [file Table_7.DOCX]

**Supplementary Table 7.** Results of forward selection based on the 15 AFLP, 4 MSAP-m, and 13 MSAP-u outliers potentially evolved under selection. Environmental variables were classified into three categories, i.e., bioclimate, ecology, and topology, and analyzed separately.

|  | Environmental variable |  | *R*^2^ | Adjusted *R*^2^ | *F* (*P*) |
| --- | --- | --- | --- | --- | --- |
| AFLP |  |  |  |  |  |
| Bioclimate | BIO4 |  | 0.331 | 0.324 | 49.42 (0.001) |
|  | BIO15 |  | 0.076 | 0.071 | 12.68 (0.001) |
| Ecology | NDVI |  | 0.204 | 0.196 | 25.67 (0.001) |
|  | PET |  | 0.024 | 0.016 | 3.06 (0.006) |
| Topology | Aspect |  | 0.149 | 0.143 | 19.33 (0.001) |
|  | Slope |  | 0.088 | 0.079 | 9.63 (0.001) |
| MSAP-m |  |  |  |  |  |
| Bioclimate | BIO4 |  | 0.318 | 0.311 | 46.70 (0.001) |
|  | BIO15 |  | - | - | - |
| Ecology | NDVI |  | 0.109 | 0.100 | 12.2 (0.001) |
|  | PET |  | 0.033 | 0.025 | 3.82 (0.007) |
| Topology | Aspect |  | 0.140 | 0.132 | 17.02 (0.001) |
|  | Slope |  | 0.052 | 0.043 | 5.49 (0.010) |
| MSAP-u |  |  |  |  |  |
| Bioclimate | BIO4 |  | 0.280 | 0.273 | 38.85 (0.001) |
|  | BIO15 |  | 0.044 | 0.038 | 6.48 (0.001) |
| Ecology | NDVI |  | 0.158 | 0.150 | 18.83 (0.001) |
|  | PET |  | 0.044 | 0.037 | 5.49 (0.002) |
| Topology | Aspect |  | 0.152 | 0.146 | 19.80 (0.001) |
|  | Slope |  | 0.086 | 0.077 | 9.42 (0.001) |

*BIO4, monthly temperature variation; BIO15, monthly precipitation variation; NDVI, normalized difference vegetation index; PET, potential evapotranspiration.*

*The environmental variables are ranked by their contribution to variation according to the adjusted-R^2^ estimated using redundancy analysis.*
