# Supplementary material for: The Contribution of Neutral and Environmentally Dependent Processes in Driving Population and Lineage Divergence in Taiwania (Taiwania cryptomerioides)
Source: Front Plant Sci. 2018 Aug 8;9:1148. doi: 10.3389/fpls.2018.01148 (PMC6092574; doi:10.3389/fpls.2018.01148)
Supplement: Supplementary Table 8 — The relative importance of the environmental variables acting as selective drivers of potentially genetic and epigenetic variations evaluating using AICc values (ΔAICc ≤ 3) using R package MuMIn. The models listed in each analysis are a 95% confidence set identified on the basis of AICc. Relative importance denotes the probability that a given environmental variable, among all parsimonious models considered, is in the model best approximating to the best model. All bold predictor variables have confidence intervals that do not include zero. [file Table_8.DOCX]

**Supplementary Table 8.** The relative importance of the environmental variables acting as selective drivers of potentially genetic and epigenetic variations evaluating using AICc values (∆AICc ≤ 3) using R package MuMIn. The models listed in each analysis are a 95% confidence set identified on the basis of AICc. Relative importance denotes the probability that a given environmental variable, among all parsimonious models considered, is in the model best approximating to the best model. All bold predictor variables have confidence intervals that do not include zero.

|  | Relative importance | | | | | |  | Best model | | |  |
| --- | --- | --- | --- | --- | --- | --- | --- | --- | --- | --- | --- |
|  | BIO4 | BIO15 | NDVI | PET | Aspect | Slope |  | AICc | logLik | Weight |  |
| AFLP |  |  |  |  |  |  |  |  |  |  |  |
| aP2_204 | **0.75** | 0.43 | 0.39 | 0.16 | 0.59 | 0.28 |  | 118.0 | -54.81 | 0.506 |  |
| aP5_139 | **0.94** | 0.55 | 0.24 | 0.14 | 0.15 | 0.20 |  | 94.1 | -42.85 | 0.680 |  |
| aP5_168 | **1.00** | 0.44 | 0.21 | 0.12 | 0.24 | 0.25 |  | 105.6 | -48.59 | 0.588 |  |
| aP9_133 | 0.24 | **1.00** | **1.00** | **1.00** | **1.00** | **1.00** |  | 103.9 | -44.37 | 0.765 |  |
| aP9_322 | **1.00** | 1.00 | 0.16 | 0.22 | 0.26 | 0.18 |  | 78.9 | -35.23 | 0.671 |  |
| aP13_142 | **0.89** | 0.14 | 0.31 | 1.00 | 0.11 | 0.34 |  | 103.9 | -47.73 | 0.649 |  |
|  |  |  |  |  |  |  |  |  |  |  |  |
| MSAP-m |  |  |  |  |  |  |  |  |  |  |  |
| mP9MH_214 | **0.73** | 0.38 | 0.57 | 0.53 | 0.63 | 0.48 |  | 108.8 | -47.97 | 0.509 |  |
| mP16MH_198 | **1.00** | 0.16 | 0.15 | 0.13 | 0.11 | 0.12 |  | 107.6 | -50.69 | 0.664 |  |
|  |  |  |  |  |  |  |  |  |  |  |  |
| MSAP-u |  |  |  |  |  |  |  |  |  |  |  |
| uP14MH_102 | 0.25 | 0.41 | **1.00** | 0.16 | 0.33 | 0.17 |  | 86.0 | -39.81 | 0.544 |  |
| uP15MH_134 | **0.96** | 0.44 | 0.29 | 0.22 | 0.50 | 0.33 |  | 113.1 | -51.26 | 0.527 |  |
