# Supplementary material for: The Contribution of Neutral and Environmentally Dependent Processes in Driving Population and Lineage Divergence in Taiwania (Taiwania cryptomerioides)
Source: Front Plant Sci. 2018 Aug 8;9:1148. doi: 10.3389/fpls.2018.01148 (PMC6092574; doi:10.3389/fpls.2018.01148)
Supplement: Supplementary Table 9 — Linkage disequilibrium between outlier genetic and epigenetic loci using a two locus exact test implemented in ARLEQUIN, and significance determined by 10,000 permutations. [file Table_9.DOCX]

**Supplementary Table 9.** Linkage disequilibrium between outlier genetic and epigenetic loci using a two locus exact test implemented in ARLEQUIN, and significance determined by 10,000 permutations.

|  | Lineages | | |
| --- | --- | --- | --- |
| Outliers | Chinese | Taiwanese | Vietnamese |
| 1: aP1_264 |  |  | 4***, 17*, 18*, 20*, 28*** |
| 2: aP1_377 |  |  | 5**, 28* |
| 3: aP2_195 |  | 8**, 9*, 11*, 13*, 14**, 21* | 7*, 8*, 9***, 22*, 25* |
| 4: aP2_204 |  | 6** | **28***, 32* |
| 5: aP4_287 |  | 11***, 20* |  |
| 6: aP5_139 |  | **18***, 27* | 16*, 17*, 27* |
| 7: aP5_168 |  | 11** | 31* |
| 8: aP9_133 |  | 10*, 14*, 21**, **24*** | 13**, 17**, **18***, 20*, 25*** |
| 9: aP9_322 |  | 13*, 15**, **18***, 21* | 11***, **28***** |
| 10: aP9_391 |  |  |  |
| 11: aP12_243 | 25*, 30* | 13* |  |
| 12: aP13_142 |  | 15**, 31* | 13**, 31* |
| 13: aP13_160 |  | 14**, 21* |  |
| 14: aP13_235 |  | 21* | 22** |
| 15: aP13_285 |  | 21* |  |
| 16: mP7MH_201 | 22**, 27*, 31*** | 18**, 21***, 22*, 24**, 30* |  |
| 17: mP9MH_207 | 31*, 32* | 30** | 19**, 20***, 28*, 32*** |
| 18: mP9MH_214 |  | 19*, 22**, 26***, 28*, 30***, 31*** | 20***, 22*, 26**, 29*, 31* |
| 19: mP16MH_198 |  | 26**, 30** | 20*, 28*, 29* |
| 20: uP5MH_169 |  | 26*, 31*** | 28*, 29**, 30* |
| 21: uP6MH_135 |  | 27**, 30* |  |
| 22: uP9MH_158 |  |  | 32* |
| 23: uP13MH_117 | 31* |  |  |
| 24: uP14MH_102 |  | 26*, 30* |  |
| 25: uP14MH_209 | 29* |  | 26*, 31**, 32** |
| 26: uP14MH_255 |  | 30**, 31** | 29**, 31** |
| 27: uP15MH_106 | 31* |  | 30*, 32* |
| 28: uP15MH_134 |  |  | 31* |
| 29: uP15MH_227 | 30* | 32* |  |
| 30: uP16MH_169 |  |  |  |
| 31: uP16MH_248 |  |  | 32* |
| 32: uP16MH_339 |  |  |  |

*Number in bold face and underlined indicate significant linkage disequilibrium between AFLP and MSAP loci that were revealed to be strongly associated with environmental variable(s) using MuMIn (Table 6).*

*, *P* < 0.05

**, *P* < 0.01

***, *P* < 0.001
